# Structural and biophysical studies of human E3 ligase Deltex 4

**DOI:** 10.1042/BCJ20260406

**Published:** 2026-10-27

**Authors:** Milan M. Fowkes, Jonathan M. Elkins

**Affiliations:** Centre for Medicines Discovery, Nuffield Department of Medicine, University of Oxford, NDM Research Building, Old Road Campus, Oxford, OX3 7FZ, U.K.

**Keywords:** Deltex, Deltex 4, DTX4, E3 ligases, ubiquitin ligases

## Abstract

Deltex (DTX) E3 ligases such as DTX1 and DTX2 are known to bind to ADP-ribosylated substrates and consequently ubiquitinate them. However, little is known about the role of the DTX E3 ligase DTX4 in substrate binding and ubiquitination. Here, we present the crystal structure of the DTX4 RING-DTC domains in complex with ADP-ribose to a final resolution of 2.8 Å and uncover a unique domain swap within the RING domain. Structural comparison with the closely related RING-DTC domains of DTX1 and DTX2 reveals similarities but also differences in the mode of ligand binding and the number and type of conserved residues. Biophysical assays to assess the strength of binding to nucleotide units of poly-ADP-ribose (PAR) demonstrate a preference for binding to ADP-ribose, strengthening existing evidence for PAR binding. Collectively, the structural and binding data offer insights into the active structure of DTX4 and its interaction with nucleotide units of PAR.

## Introduction

Ubiquitination is a post-translational modification that regulates many cellular processes [1]. Canonical ubiquitination is completed by E3 ligases, which catalyse the terminal step of the enzymatic cascade by attaching a ubiquitin molecule to a lysine on the target protein [1,2]. Ubiquitination of target proteins can have a wide range of effects: from monoubiquitination that can result in repair of DNA damage, to polyubiquitination via Lys48 of ubiquitin that marks the protein for degradation by the proteasome [3,4]. The deltex E3 ubiquitin ligase 4 (DTX4) is a member of the Deltex (DTX) family of E3 ligases [5], and participates in key developmental and immune pathways, such as Notch signalling [6] and type I interferon signalling [7]. In the former, it ubiquitinates Notch1 at the cell surface following ligand binding, resulting in bilateral endocytosis of the Notch1-DTX4 fragment and the Notch1 extracellular domain by the Notch1- and ligand-expressing cells, respectively [6]. The Notch1 fragment is then degraded in endocytic vesicles by a disintegrin and metalloproteinase domain-containing protein 10, generating the Notch intracellular domain that goes on to control gene expression [6]. In the latter pathway, the TANK binding kinase 1 (TBK1) is polyubiquitinated by DTX4 at Lys670 following recruitment by the cytoplasmic protein NLR family pyrin domain containing 4 (NLRP4) [7]. This degradation of TBK1 through negative regulation by NLRP4 ensures that homeostasis is maintained in the innate immune system following infection by viruses [7]. A recent study using mass spectrometry to analyse pulldowns of whole-cell lysates from cells expressing the C-terminal domain of DTX4 [8] revealed potential interaction partners including those in DNA damage repair (helicase like transcription factor) [9], protein degradation (proteasome 20S subunit beta 3) [10] and the E3 ligase DTX3 [8].

In the context of disease, DTX4 is associated with several malignancies [11–13]. For example, microarray gene expression profiling showed a higher expression of DTX4 mRNA in murine melanoma cancer stem-like cells [11] and in patients with nasopharyngeal carcinoma when compared with controls [12]. In addition to significantly elevated expression of DTX4 mRNA in human thyroid cancer cell lines and tissues, knockdown of DTX4 in a xenograft mouse model of thyroid cancer significantly reduced tumour growth compared with the control [13].

Structurally, DTX4 comprises tandem N-terminal WWE domains coupled with a C-terminal Really Interesting New Gene (RING)-H2 domain and a Deltex C-terminal (DTC) domain, similar to the DTX family members DTX1 and DTX2 (Figure 1A).

**Figure 1:**
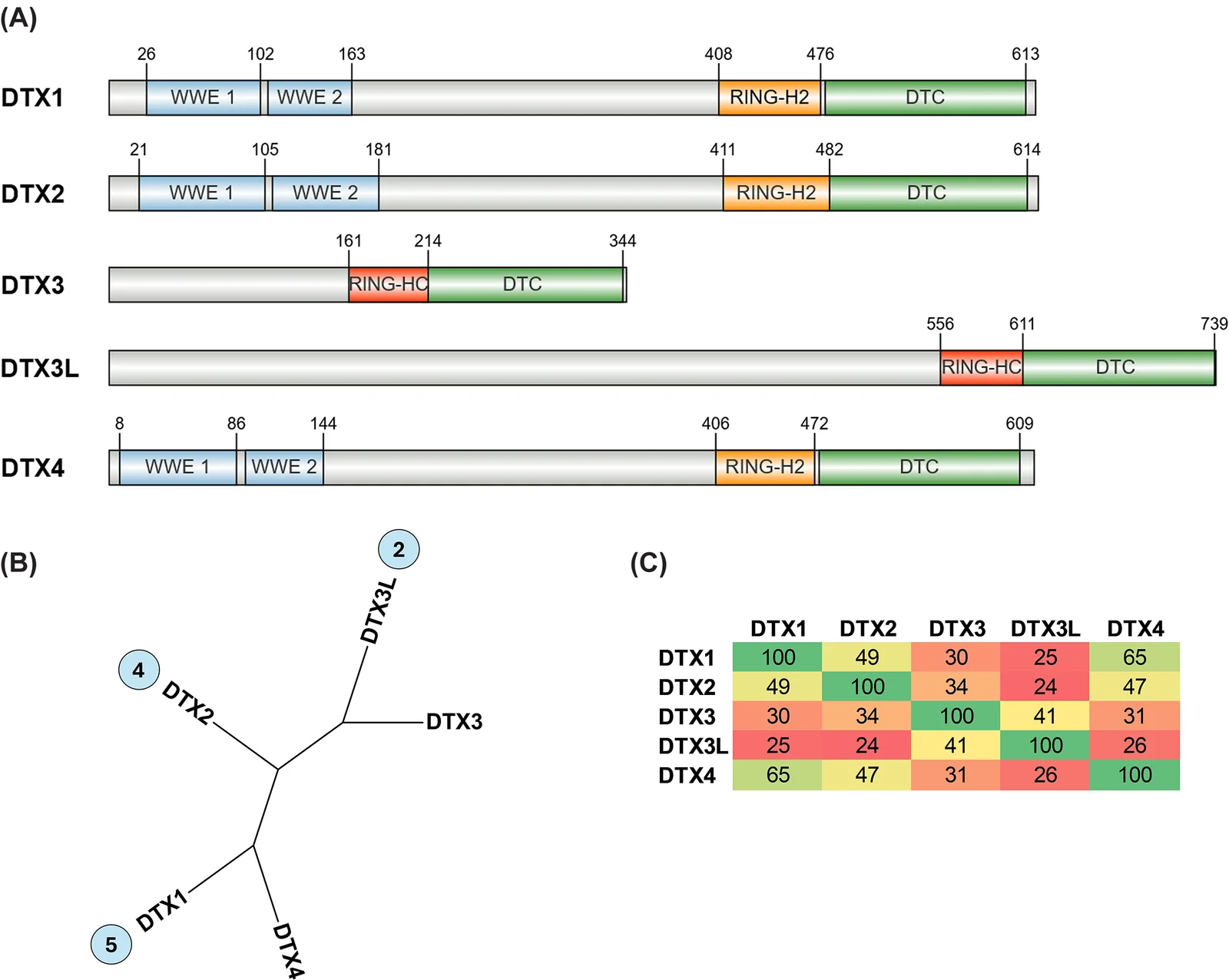
Domain organisation and phylogeny of the human DTX E3 ligases (**A**) Domain architecture of the human DTX E3 ligases, drawn to scale using IBS 2.0 [14]. DTX = Deltex, RING = Really Interesting New Gene; DTC = Deltex C-terminal domain. (**B**) Phylogenetic tree of human DTX E3 ligases. Full-length protein sequences were obtained from the NCBI protein database and aligned using Clustal Omega Multiple Sequence Alignment via the EMBL-EBI Job Dispatcher tool [15]. Trees were produced using IQ-TREE 3 [16] and visualised using iTOL [17]. Circles with numerical values represent the number of structures for each ligase in the PDB as of September 2026. (**C**) Percentage identity matrix for full-length human DTX E3 ligases. The colour-coded percentage identities were generated from the multiple sequence alignment performed in (**B**).

Phylogenetically however, it is most closely related to DTX1 (Figure 1B) with 65% identity compared with 47% for DTX2 when comparing full-length forms (Figure 1C). More recently, DTX4 was shown to ADP-ribosylate ubiquitin at Gly76 *in vitro* through the RING-DTC domains, a mechanism that is shared with all other members of the DTX family [18]. The DTX E3 ligase DTX2 has been studied extensively in the context of its role in substrate ubiquitination [8,19–22]. Mechanistically, the DTC domain of DTX2 binds ADP-ribosylated substrates whose ubiquitination is facilitated by the RING-H2 domain [8,19,20]. In addition, the WWE domains of DTX2 have been shown to play a key role in recruiting DTX2 to DNA lesions through binding to ADP-ribosylated substrates, with support from the RING-DTC domains [23,24].

Similarly, DTX1 has been shown to ubiquitinate poly-ADP-ribose (PAR) at the adenosine terminus [25] and has also been localised to sites of DNA double-strand breaks via WWE domain binding to PAR [24]. Although DTX4 has been shown to bind PAR through its DTC domain in whole-cell lysate pulldown assays, this interaction has not been investigated further [8]. Furthermore, despite the existence of crystal structures for the DTX1 RING-DTC [18] and WWE domains [26] as well as the DTX2 RING-DTC domains [8], no experimental protein structures for DTX4 have been published to date.

Herein, we describe the X-ray crystal structure of the RING-DTC domains of DTX4, revealing a unique domain swap within the RING domain. We compare the DTX4 RING-DTC structure to those of DTX1 and DTX2 and analyse the binding of DTX4 to components of PAR (i.e. ADP and ADP-ribose) using isothermal titration calorimetry (ITC). Taken together, the data support a similar structural mechanism for substrate ubiquitination by DTX4 compared with the previously characterised DTX1 and DTX2.

## Results

### Expression and purification of DTX4 RING-DTC domains

To enable structural studies of the DTX4 RING-DTC domains, we cloned DNA corresponding to truncations of human DTX4 into plasmids for overexpression in *E. coli*. The resulting constructs consisted of an N-terminal hexahistidine (His6) tag, a Tobacco Etch Virus (TEV) protease recognition site (to allow removal of the tag), and the DTX4 RING-DTC domain. We evaluated multiple different truncations around the RING-DTC domains (Supplementary Table S1), of which two expressed the target protein. Litre-scale expression of the DTX4 construct spanning residues Gln383 to Asp619 (Figure 2A) and subsequent purification (Supplementary Figure S1B) yielded protein suitable for crystallisation trials.

**Figure 2:**
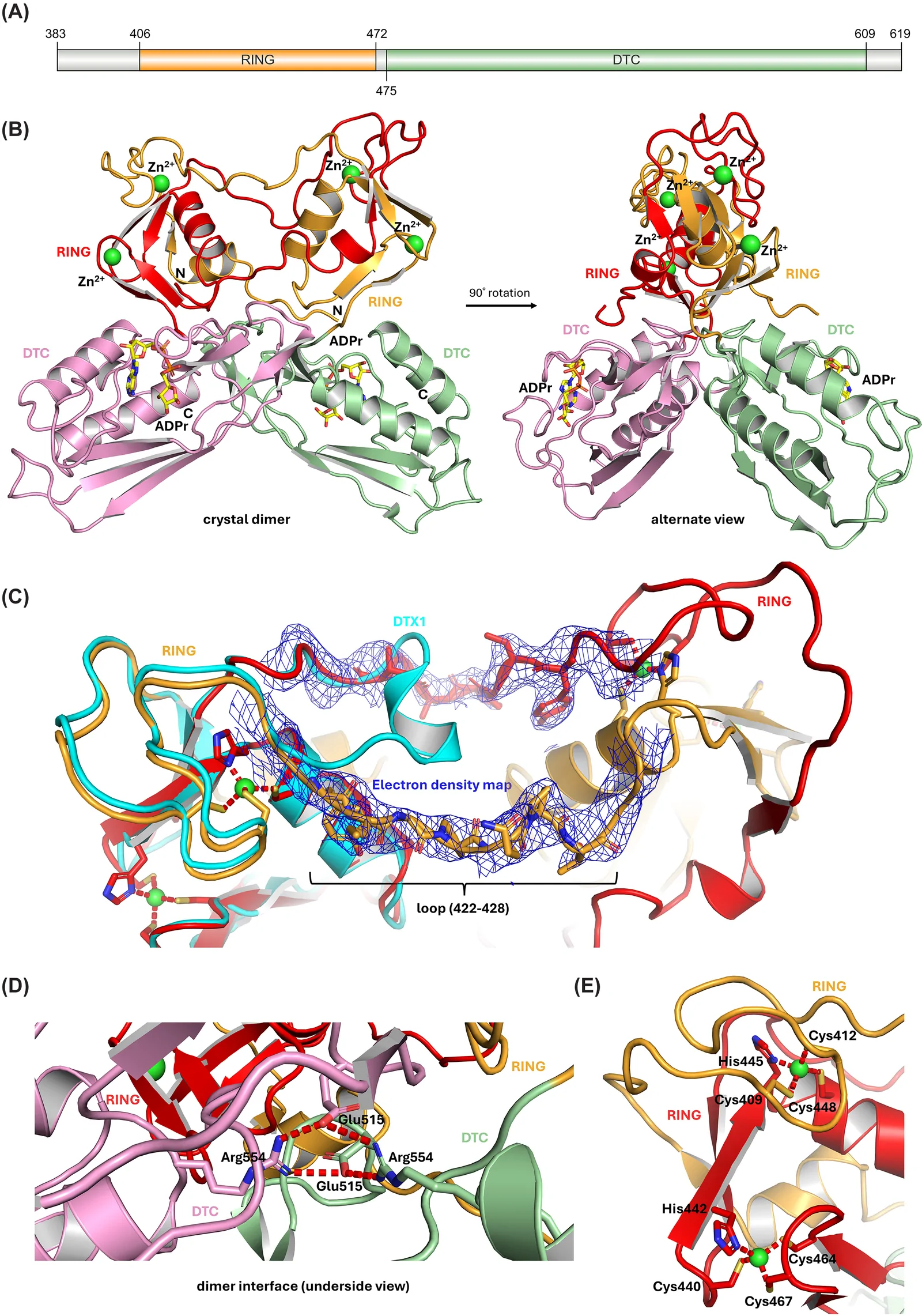
The Crystal structure of DTX4 RING-DTC in complex with ADP-ribose (**A**) Domain architecture of the DTX4 RING-DTC domains construct used for crystallisation. Domain boundaries were defined by the NCBI Conserved Domains Database [27]. (**B**) The structure of the DTX4 RING-DTC dimer as seen in the crystal structure at two different viewing angles. The crystallographic asymmetric unit contains two copies of the RING-DTC domain, which form dimers by crystallographic symmetry around a two-fold rotation axis. The RING and DTC domains of one monomer are coloured light orange and green as in (**A**), and the RING and DTC domains of the other monomer are coloured red and pink, respectively. ADP-ribose is depicted as sticks with yellow carbon atoms. The conserved zinc ions found in the RING domains are shown as green spheres. (**C**) The domain swap occurs at a loop that is two amino acids shorter in DTX4 than in DTX1. The structure of the RING domain of DTX1 is shown in light blue superimposed on the RING domain of DTX4. A 2*F*_o_-*F*_c_ experimental electron density map (dark blue) is shown covering residues 420–430 of DTX4. (**D**) Detail of the interface between the DTC domains of both monomers, with hydrogen bonds shown as dotted red lines. (**E**) Detail of the binding of zinc atoms in the RING domains. The first zinc binding site (top) is formed by two cysteines from one DTX4 monomer and a cysteine and a histidine from another monomer. The second zinc binding site (bottom) is contained within the same DTX4 monomer.

### The crystal structure of DTX4 RING-DTC bound to ADP-ribose reveals a domain swap at the RING domain

Given that the RING-DTC domains of DTX1 and DTX2 have been successfully crystallised in complex with nucleotide ligands (i.e. with NAD^+^ [18] and ADP-ribose [8], respectively), we attempted crystallisation of the DTX4 RING-DTC domains with ADP and ADP-ribose. Ultimately, DTX4 in complex with ADP-ribose formed crystals that diffracted X-rays sufficiently to solve the structure of the RING-DTC domains to a resolution of 2.8 Å (Figure 2B and Supplementary Table S2).

We were able to solve the crystal structure by molecular replacement using the structure of DTX1 bound to NAD^+^ [18] as a search model. However, during the process of solving the structure it became apparent that the DTX4 protein was in a domain-swapped form, with the N-terminal portion of the RING domain (residues 383–428) bound to an adjacent DTX4 molecule in the crystal lattice. The effect within the crystal is that each RING domain is composed of residues 383–428 from one DTX4 molecule and residues 429–619 of another (Figure 2B). The formation of the dimer is made possible by DTX4 possessing a loop (residues 422–428) that is two amino acids shorter than the equivalent loop in DTX1, which contains two additional hydrophobic residues: Val and Leu (Figure 2C and Supplementary Figure S2A). Superposition of the DTX4 RING domain with the DTX1 RING domain (PDB: 6Y5N) shows the effect of the domain swap on the conformation of the N-terminal region of the RING domain (Supplementary Figure S2B). The N-terminal residues (383–428) of DTX4 form an elongated conformation that allows them to bind the adjacent DTX4 molecule in the structure, with one Zn^2+^ ion coordinated by residues from two different DTX4 molecules. To analyse whether the shorter loop in DTX4 may promote a domain swap, we generated a structural model of the DTX4 RING domain that included the Val-Leu residues from DTX1 in the 422–428 loop using the AlphaFold 3 server (ipTM = 0.91, pTM = 0.82) [28]. This chimeric model superposes onto the DTX1 RING domain with a root mean square deviation (RMSD) of 0.725 Å over 343 Cα atoms (Supplementary Figure S2C). Both RING domains showed an essentially identical fold. We then generated a structural model of the wild-type DTX4 RING domain without these residues and superposed this onto the DTX1 RING domain as before (an RMSD of 0.506 Å over 336 Cα atoms, Supplementary Figure S2D). Within the limitations of the AlphaFold model, this revealed that the additional Val-Leu residues do not affect the fold of the predicted DTX4 RING domain. DTX4 can therefore form a canonical monomeric RING domain structure both with and without these additional residues.

While testing various crystals of DTX4 for diffraction, we observed that all contained the same unit cell and symmetry, suggesting that the domain-swapped dimer had pre-formed in solution. The temperature factors of the domain-swapped region were not significantly higher than the rest of the structure (Supplementary Figure S3). Although the dimer interface seen in the structure suggests hydrogen bonding between pairs of residues in the DTC domains (Glu515 and Arg554), there is little DTC-DTC domain interaction compared with the substantial RING-RING interactions. In addition, no significant contacts were observed between the RING domain of one protomer and the DTC domain of another (Figure 2D). The dimerisation interface also included one of the conserved zinc atoms found in the RING domains (Figure 2E). This zinc atom was bound by two cysteines from one DTX4 monomer (Cys409 and Cys412) and a cysteine (Cys448) and a histidine (His445) from the other DTX4 monomer. Overall, the DTX4 RING-DTC domains exist in the crystal as a dimer formed by the extensive RING domain interactions (Figure 2B,E).

Domain swaps in crystal structures have been observed previously and may in some cases be related to the function of the protein. For example, exchange of the activation loops of several protein kinases was shown to be linked to their *trans*-activation, and the proteins were shown to be dimeric in solution [29]. We were therefore interested in determining whether the dimeric form of the DTX4 RING-DTC domains observed in the crystal structure was present in solution. We suspected that the RING-DTC domains may not dimerise at moderate concentrations (<100 μM), as no evidence of dimerisation was observed during purification of the protein by size-exclusion chromatography (SEC; data not shown). We injected concentrated DTX4 protein (1 μl, 860 μM) from the same batch used for crystallisation onto an analytical SEC column and compared the elution volume to a set of protein standards of known molecular weight (Supplementary Figure S4). The 26 kDa DTX4 RING-DTC domains eluted at the expected volume for a monomer, with the elution peak appearing at an elution volume between those for the 17 kDa and 45 kDa molecular weight standards. Further injections using higher volumes (5 μl or 10 μl) at the same concentration simply resulted in broadening of the elution peak (data not shown). It therefore appears that at analytical SEC concentrations of up to 32 μM the protein is monomeric, while at the higher concentrations achieved during the vapour diffusion crystallisation process dimerisation is possible.

However, given the potential contribution of the DTC domains towards the dimer interface, we also performed analytical SEC in the presence of ADP-ribose. We injected a series of increasing volumes of concentrated DTX4 (731 μM) with ADP-ribose (1.5 mM) in buffer containing ADP-ribose (Supplementary Figure S5). The DTX4 protein had a similar elution time to that in the absence of ADP-ribose, indicating that complexation with this nucleotide did not cause dimerisation under these conditions. We cannot, however, rule out that dimerisation may have been promoted by the effects of buffer composition in the crystallisation experiment or that it could be promoted by other factors present in a cellular environment and therefore have functional relevance.

### A domain-swapped DTX4 RING domain could still engage in catalysis of ubiquitination

To investigate the potential impact of the domain swap on the binding of DTX4 to an E2∼Ub complex, we aligned the DTX4 RING domain (residues 443–474) onto the RING domain of RNF38:UbcH5B∼Ub (residues 423–458, PDB: 4V3L), a stable RING E3 ligase:E2∼Ub mimic complex [30] (Figure 3A). This showed that the domain-swapped DTX4 RING domain does not occlude the E2 binding surface. The more compact part of the DTX4 RING domain (with a single zinc ion) occupies the same space as the RNF38 RING domain and interacts with the E2 enzyme as well as ubiquitin. The less compact and more extended part has minimal interaction with the E2 binding surface, except via the N-terminal tail. In addition, the DTX4 RING domain does not interfere with the orientation of the ubiquitin donor, as the isopeptide bond (which mimics the thioester of E2∼Ub) is located away from the main interaction surface (Figure 3B, black dotted circle).

**Figure 3:**
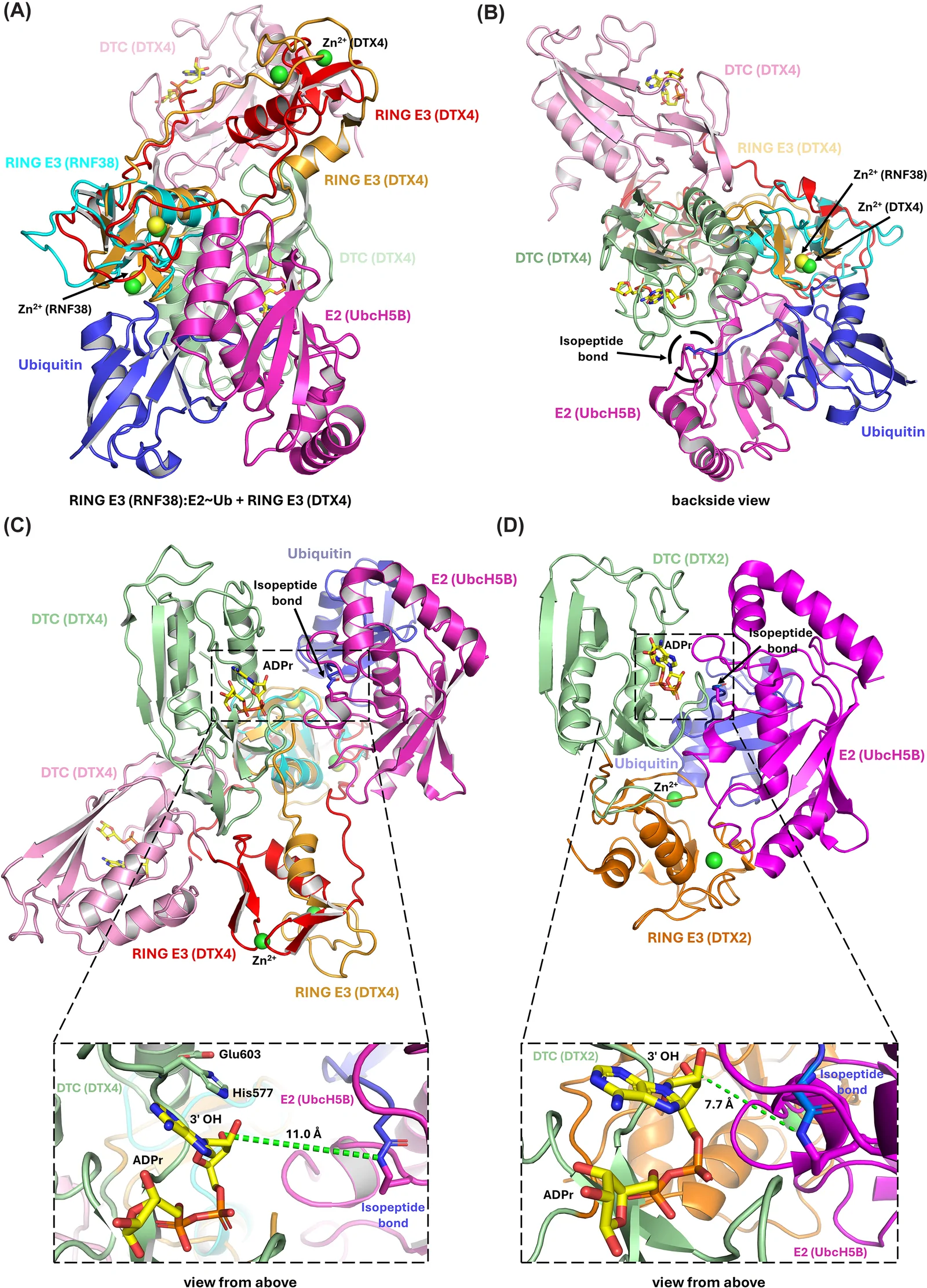
Analysis of the potential for a dimeric DTX4 RING-DTC domain to form a productive E3 ligase:E2∼Ub complex (**A,B**) The DTX4 dimer structure was superimposed on the RNF38:UbcH5B∼Ub mimic structure (PDB: 4V3L) using only the coordinates of the RING domains (residues 443–474 and 423–458, respectively) for the superimposition. The RING domains of DTX4 and RNF38 are coloured gold/red and cyan, respectively, with the E2 enzyme UbcH5B coloured in magenta and ubiquitin in blue. The DTC domains of DTX4 are coloured pink/green. Zinc ions are shown as green (DTX4) or yellow (RNF38) spheres. (**C,D**) Alignment of the DTX4 RING domain (residues 443–474) and DTX2 RING domain (residues 448–482) with the RING domain of the RNF38:UbcH5B∼Ub mimic (residues 423–458) showing the position of the DTC domains of DTX4 and DTX2 (both green) with details of the active site shown in each inset (dotted black rectangles). The ADP-ribose and the isopeptide bond are shown as sticks with yellow or blue carbon atoms, respectively. The dotted green line in each inset indicates the distance between the 3′ OH on ADP-ribose and the amide NH of the isopeptide bond, viewed from above.

Comparing the DTX4 DTC domain in the context of this RING domain alignment (Figure 3C) we discovered that the 3′ OH of the ADP-ribose acceptor was located 11.0 Å from the isopeptide bond of the ubiquitin donor (Figure 3C inset, dotted green line, measured from the amide NH). By contrast, alignment of the RING domain of DTX2 RING-DTC (residues 448–482, PDB: 6Y3J) with RNF38:UbcH5B∼Ub on the RING domain (residues 423–458) gave a distance of 7.7 Å, which is 1.4-fold shorter between acceptor and donor (Figure 3D inset, dotted green line). From this analysis it is possible that the domain swap in the DTX4 RING domain leads to a more extended conformation that places the DTC domain further from the ubiquitin donor and therefore hinders or reduces the rate of thioester bond formation between the nucleotide and the E2∼Ub mimic. However, there is significant flexibility in the relative positions of the RING and DTC domains, and the difference in distance is likely strongly influenced by crystal packing. Therefore, a catalytically productive dimeric DTX4 remains a possibility.

DTX4 His577 (2.7 Å) and Glu603 (2.6 Å) are close to the 3′ OH of ADP-ribose (Figure 3C inset), and by analogy to DTX2 are part of the catalytic triad for hydroxyl ubiquitination [19]. In this mechanism, Glu603 would hydrogen bond to His577 which would then deprotonate the 3′ hydroxyl group of the ribose ring prior to nucleophilic attack on the thioester [19]. In DTX2, mutation of the equivalent residues (Glu608 and His582, respectively) abrogated ubiquitination of NAD^+^ [19]. It therefore appears that a similar mechanism could work for DTX4, certainly in the monomeric form, but also in the dimeric form assuming sufficient flexibility of the quaternary structure.

### Comparison of the DTX4 RING-DTC and DTX1/DTX2 RING-DTC domains

The DTX4 structure reveals the expected arrangement of the RING and DTC domains and the ADP-ribose ligand bound to the DTC domain (Figure 4A). In the nucleotide-binding site of the DTC domain (Figure 4B), there is hydrogen bonding between the phosphate groups of ADP-ribose and the side chains of residues Ser563, Ser562, Thr564, and Arg530 as well as between the endocyclic oxygen and the 1′ hydroxyl group and Arg530. In addition, the adenine ring forms two hydrogen bonds with Thr580 while the 2′ and 3′ hydroxyl groups of ribose hydrogen bond to His577.

**Figure 4:**
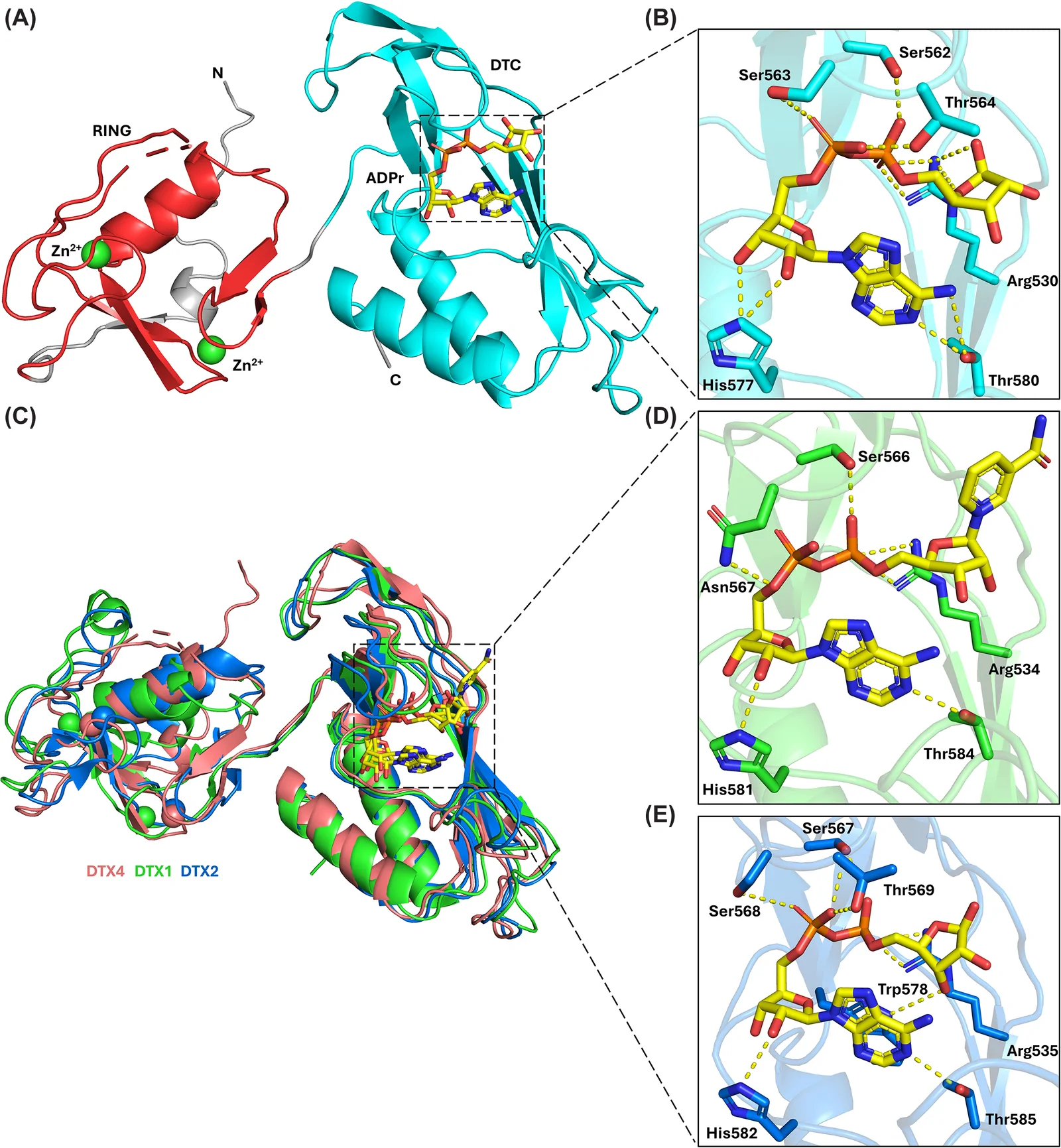
The DTX4 RING-DTC domains in complex with ADP-ribose compared with the nucleotide-bound DTX1 and DTX2 RING-DTC domains For ease of viewing, the DTX4 structure is presented as a monomer, as if the domain-swapped dimer was not present, by combining atomic coordinates of the relevant parts of the dimer present in the crystals. In all panels, ADP-ribose is depicted as sticks with yellow carbon atoms. Zinc ions are shown as spheres and hydrogen bonds are indicated by dotted yellow lines. (**A**) Structure of the DTX4 RING-DTC domains. The RING and DTC domains are coloured red and cyan, respectively. (**B**) The ADP-ribose binding site in the DTC domain of DTX4. (**C**) Overlay of the RING-DTC domains of DTX4 (salmon pink), DTX1 (green, PDB: 6Y5P) and DTX2 (blue, PDB: 6Y3J). (**D**) The NAD^+^ binding site in the DTC domain of DTX1. (**E**) The ADP-ribose binding site in the DTC domain of DTX2.

An overlay of the DTX4 RING-DTC domain structure with the corresponding structures of DTX1 and DTX2 revealed a high level of structural conservation, including the relative positions of the RING and DTC domains and location of the nucleotide-binding site (Figure 4C).

The nucleotide-binding site showed that hydrogen bonding interactions between ADP-ribose and His577, Thr580 and Arg530 of DTX4 were conserved in DTX1 and DTX2 (Figure 4D,E, respectively). However, DTX4 Ser562 is only conserved in DTX1, which also has an additional hydrogen bonding interaction between DTX1 Asn567 and the phosphate oxygen of NAD^+^. In contrast, DTX2 retained the hydrogen bonding interactions between ADP-ribose and the Ser and Thr residues but contained an additional hydrogen bonding interaction between Trp578 and the 3′-hydroxyl group of ribose, made possible by the different conformation of the ribose ring in the binding site (Figure 4E).

### DTX4, DTX1, and DTX2 preferentially bind ADP-ribose

To better understand the binding of nucleotide ligands to the isolated DTC domain of DTX4, we expressed this domain in *E. coli* and purified the protein for biophysical studies (Supplementary Table S3 and Supplementary Figure S6). We then analysed its binding to ADP-ribose and ADP using ITC (Figure 5A). The DTX4 DTC domain bound ADP-ribose with a *K*_D_ of 44.9 μM. While no comparable data for other isolated DTC domains have been reported to date, this value is in a functionally relevant range for binding to ADP-ribosylated targets in cells [26,31]. Conversely, we did not detect binding of the DTC domain to ADP. In addition, we did not observe a significant heat of dilution when injecting ADP-ribose into buffer or buffer into buffer (Supplementary Figure S7).

**Figure 5:**
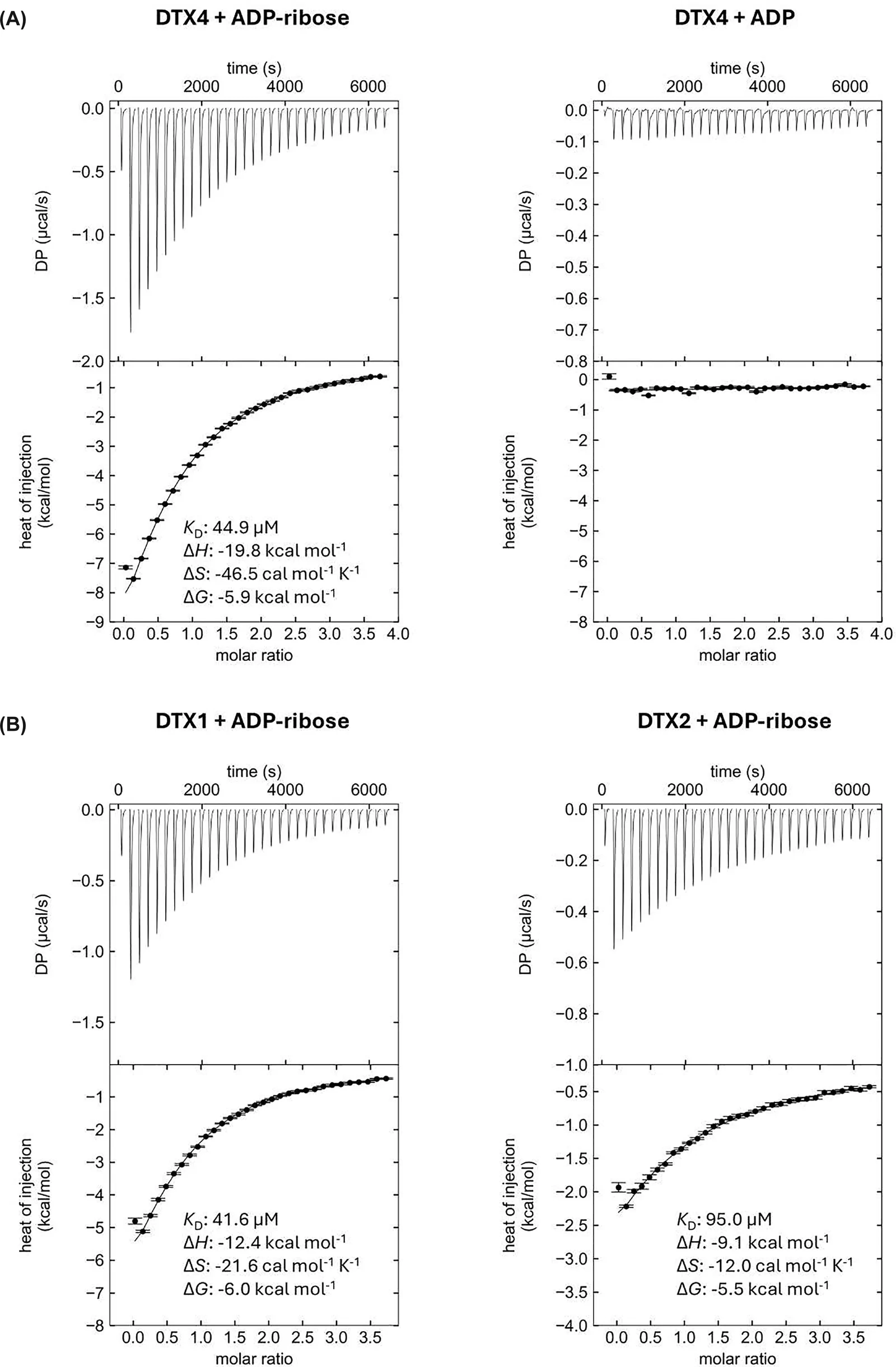
DTC domain binding to nucleotide ligands ITC data for (**A**) DTX4 DTC domain binding to ADP-ribose and ADP and (**B**) DTX1 and DTX2 DTC domain binding to ADP-ribose at 298 K. Data were processed with NITPIC [32,33] fitted to a one-site binding model using SEDPHAT [34] and visualised with Gussi [35].

To directly compare the ability of DTX4, DTX1 and DTX2 to bind ADP-ribose, we also expressed and purified the DTC domains of DTX1 and DTX2 (Supplementary Table S4 and Supplementary Figure S8). As for DTX4, no binding to ADP was observed for either DTX1 and DTX2 (Supplementary Figure S9). In contrast, ADP-ribose bound to DTX1 with a similar affinity to DTX4 (*K*_D_ = 41.6 μM), but to DTX2 with approximately two-fold lower affinity (*K*_D_ = 95.0 μM) (Figure 5B). The stronger binding of DTX4 to ADP-ribose compared with DTX2 is supported by the change in binding enthalpy (Δ*H*) and binding entropy (Δ*S*). Although the entropy change is more negative for ADP-ribose binding to DTX4 compared with DTX2 (cf. Δ*S* = -46.5 cal mol^−1^ K^−1^ to -12.0 cal mol^−1^ K^−1^ for DTX2), this entropic penalty is outweighed by the stronger enthalpic contribution to binding (cf. Δ*H* = −19.8 kcal mol^−1^ to −9.1 kcal mol^−1^ for DTX2). Overall, this leads to a more negative Gibbs free energy of binding (Δ*G* = −5.9 kcal mol^−1^ versus −5.5 kcal mol^−1^ for DTX2) and thus lower *K*_D_ for DTX4. For DTX1, the similar dissociation constant for ADP-ribose is driven less by enthalpic contributions due to the lower entropic penalty upon ligand binding (Δ*S* = −21.6 cal mol^−1^ K^−1^ versus −46.5 cal mol^−1^ K^−1^, respectively). Similarly, for the DTX4 RING-DTC domains, we observed equivalent binding to ADP-ribose (*K*_D_ = 57.4 μM) but not ADP (Supplementary Figure S10).

Taken together, the data indicate that the DTC domains of DTX4, DTX1 and DTX2 recognise and bind to ADP-ribose consistent with published data showing DTX proteins binding to mono-ADP-ribose (MAR), PAR and more broadly to ADP-ribosylated substrates [8,19,20,22–25].

## Discussion

Despite the biological importance of DTX4, such as in the ubiquitination of Notch1 and TBK1 in Notch signalling [6] and in type I interferon signalling [7], its structure has not been elucidated so far. The domain-swapped dimer that we observed for the RING domain has, to our knowledge, not been observed previously. Furthermore, a search of the Protein Data Bank for zinc atoms bound simultaneously to residues from multiple chains did not reveal any similar structures either. An N-terminal domain swap has been described for the bacterial E3 ligase IpaH9.8, which potentially forms part of a mechanism to regulate its ubiquitinase activity following damage to the host cell [36]. However, this swap is of a different nature, occurs under oxidative conditions and is characterised by a disulphide bond involving the catalytic cysteine, thereby dimerising the protein and deactivating the ligase [36]. The observation that the same crystal form (point group and unit cell) was seen for other crystals of the DTX4 RING-DTC domains suggests that the domain-swapped dimer had pre-formed in solution. It is possible that this dimerisation was promoted by the crystallisation process, either through the higher protein concentrations or through the composition of the crystallisation solution. We also cannot rule out that the observed dimer only forms with the truncated RING-DTC construct used in our experiments and that it does not form with full-length DTX4. To date, we have been unable to overexpress the full-length DTX4 protein in sufficient quantities to test this possibility.

A number of E3 ligases are regulated via dimerisation of the RING domain [37,38]. For example, the activity of the RING finger protein 4 (RNF4) is regulated by the concentration of small ubiquitin-like modifier (SUMO) protein chains [37]. RNF4 is primarily an inactive monomeric E3 ligase at a low local concentration of SUMO chains. However, an increased local concentration of SUMO chains leads to dimerisation of the RING domain and activation of RNF4 [37]. This results in ubiquitination of poly-SUMOylated proteins as well as autoubiquitination and thus degradation of RNF4, a secondary pathway to regulating its activity [37]. In contrast, tripartite motif 5α (TRIM5α) binds to the viral capsids of immunodeficiency viruses and prevents viral replication following their entry into cells [38]. The close proximity of three RING domains results in the formation of an asymmetric trimer, which consists of two RING domains that activate the E2∼Ub conjugate, while the N-terminus of the third RING domain is auto-ubiquitinated [38].

In the case of DTX4, our modelling showed that the formation of the domain-swapped RING dimer results in an increased distance between the DTC domain and the E2∼Ub mimic, caused by the extended and less compact conformation of the RING domain (Figure 3C). When analysing the potential impact of this increased distance on the proposed mechanism of hydroxyl ubiquitination (Figure 3C,D insets) [19], the distance between the 3′ OH of ribose and the thioester bond mimic was 1.4-fold greater than that for the equivalent interaction with the DTC domain of DTX2 (Figure 3D and inset). If not a function of the crystal packing, this increase in distance could be a mechanism for dimerisation of DTX4 under high local concentrations to regulate its E3 ligase activity.

To conclude, we have described the first structure of the catalytic domains of the DTX E3 ligase DTX4, discovering a distinctive domain swap within the RING domain. Collectively, the structural and biophysical data are consistent with literature data supporting the binding of the DTX4 DTC domains to PAR [8], the binding of the DTX1 and DTX2 DTC domains to PAR [8,19,25], and the DTX2 DTC domain binding to MAR [20] and suggests a potential mechanism for regulation of its E3 ligase activity in cells.

## Methods

### Expression and purification of Deltex C-terminal domain proteins

Mammalian DTX proteins were cloned into the pNIC28-Bsa4 vector (Addgene #26103) for expression in *E. coli*. Constructs contained a His6 tag and a TEV protease cleavage sequence at the N-terminus [39]. They were transformed into BL21(DE3)-R3-pRARE2 *E. coli* cells (Addgene, #26242) [39] and grown with shaking in Luria broth (Miller) supplemented with kanamycin (25 μg/ml) to an OD_600_ of 0.6–0.8 (37°C, 200 rpm). Protein expression was induced with isopropyl β-D-1-thiogalactopyranoside (0.5 mM), and the flasks were left shaking overnight (18°C, 180 rpm, 16 h). The cells were centrifuged (5000×***g***, 10 min, 4°C), the supernatant was decanted and the cell pellet was resuspended in binding buffer consisting of 50 mM HEPES, pH 7.5, 150 mM NaCl, 20 mM imidazole, pH 7.5, 0.5 mM tris(2-carboxyethyl)phosphine (TCEP) and protease inhibitor cocktail (1:1000 dilution, MedChemExpress, #HY-K0010) and frozen at −20°C prior to purification.

Cell pellets were thawed and sonicated on ice (5 min, pulses were 5 s ‘on’ and 10 s ‘off’), DNA was precipitated with 0.15% (w/v) polyethyleneimine, and the resultant mixture was centrifuged (39,810×***g***, 30 min). The clarified supernatant was purified under gravity flow by immobilised metal affinity chromatography (IMAC) using Ni Sepharose 6 Fast Flow resin (Cytiva). The protein was eluted with binding buffer using a concentration gradient of 40–250 mM imidazole. The His6 tag was removed following overnight incubation in dialysis buffer (50 mM HEPES, pH 7.5, 150 mM NaCl, and 0.5 mM TCEP) containing TEV protease (4°C, 1:50 ratio of protease:protein). A second Ni-IMAC purification was performed using binding buffer with a concentration gradient of 10–250 mM imidazole. After each purification step, fractions were analysed by SDS-PAGE, selected, combined and concentrated to <5 ml in a Vivaspin protein spin concentrator (Vivaproducts, molecular weight cut off = 10 kDa). They were then purified by SEC on a Superdex 75 16/600 column (Cytiva) in gel filtration buffer (20 mM HEPES, pH 7.5, 150 mM NaCl, and 0.5 mM TCEP). Purified protein fractions were pooled and absorbance measured at 280 nm (A_280_) using a spectrophotometer (NanoPhotometer, Implen). Protein concentrations were determined using the Beer–Lambert–Bouguer law [40] using molar extinction coefficients computed by ExPASy ProtParam, assuming reduced cystines (https://web.expasy.org/protparam/). The identities of purified DTX proteins were confirmed by electrospray ionisation mass spectrometry (Agilent 1290 Infinity II 6530 Q-TOF) and they were frozen at −80°C until needed.

### Co-crystallisation of DTX4 RING-DTC domains with ADP-ribose

DTX4 RING-DTC domain (10 mg/ml) was incubated with ADP-ribose (1.5 mM) in gel filtration buffer (20 mM HEPES, pH 7.5, 150 mM NaCl, 0.5 mM TCEP) on ice (10 min). The mixture was centrifuged (9660×***g***, 2 min, 4°C) and the supernatant dispensed into crystallisation plates (3-Lens, SWISSCI) using a Mosquito liquid handler (STP Labtech). Plates were incubated at 20°C (Rock Imager 1000, Formulatrix) with protein-to-reservoir ratios of 2:1, 1:1 and 1:2 (150 nL final volume). Crystals of DTX4 RING-DTC domains were obtained in conditions of 80 mM polyamines, 0.1 M Buffer System 1, pH 6.5, and 30% Precipitant Mix 1 (Morpheus fusion screen, well H11, Molecular Dimensions). They were mounted in mother liquor containing 30% (v/v) ethylene glycol cryoprotectant and flash-frozen in liquid nitrogen. X-ray diffraction data were then collected at Diamond Light Source, beamline I03.

### Structural determination of DTX4 RING-DTC domains bound to ADP-ribose

X-ray diffraction data were integrated using XDS [41] and merged using AIMLESS [42]. Molecular replacement was performed in Phaser [43] using the structure of the RING-DTC domain of DTX1 bound to NAD^+^ (PDB: 6Y5P) as a search model. Refinement was performed using REFMAC5 [44]. The model was validated with MolProbity [45] and deposited in the Protein Data Bank with accession code 30XN. Processing and refinement statistics are provided in Supplementary Table S2.

### Generation of DTX4 wild-type and chimera models by AlphaFold 3

The sequence for the human DTX4 RING domain was obtained from the NCBI database, with domain boundaries defined by the NCBI Conserved Domains Database [27]. Following sequence alignment with the RING domain of DTX1 (Supplementary Figure S2A), Val and Leu were added to the DTX4 sequence after Gly424, and the resultant sequence uploaded to the AlphaFold 3 server (https://alphafoldserver.com/) [28]. The chimera model was generated with the inclusion of two zinc ions, and the predicted model with the highest confidence was aligned with the DTX1 RING domain (Supplementary Figure S2C). The wild-type model was generated in the same way, but without the inclusion of Val and Leu residues (Supplementary Figure S2D).

### Analytical size-exclusion chromatography of DTX4 RING-DTC domains

An Agilent AdvanceBio SEC 200 Å column (2.1 × 150 mm PEEK, 1.9 μm particle size) was used with a 20 min runtime at a flow rate of 0.1 ml/min with gel filtration buffer (20 mM HEPES, pH 7.5, 150 mM NaCl and 0.5 mM TCEP). UV absorbance was measured at 220 and 280 nm. Protein standards (AdvanceBio SEC 300 Å, Agilent) were reconstituted in gel filtration buffer (1 ml), gently inverted several times and sonicated (5 min) prior to being injected at 5 μl. The DTX4 RING-DTC construct was injected at a volume of 1 μl at 21.8 mg/ml. In the case of co-injection with ADP-ribose, DTX4 RING-DTC construct (731 μM) was incubated with ADP-ribose (1.5 mM) at room temperature (10 min). Following centrifugation (13,539×***g***, 2 min), the supernatant was injected at different volumes (1, 5, and 10 μl), with protein standards injected at 6 μl. The same conditions were used as described above except that gel filtration buffer contained 1.5 mM ADP-ribose. Plots of UV absorbance against time (min) were used to compare the retention times between the DTX4 protein and the protein standards to estimate the molecular weight of the DTX4 RING-DTC domains in solution.

### Determination of nucleotide ligand binding by isothermal titration calorimetry

The binding of endogenous nucleotide ligands (ADP and ADP-ribose) to human DTX proteins was determined using a VP-ITC MicroCalorimeter (MicroCal) at 25°C. Purified proteins (100 μM, 1.1 ml) were dialysed overnight in 20 mM HEPES, pH 7.5, 150 mM NaCl and 0.5 mM TCEP. The dialysis buffer was filtered through a 0.22 μm vacuum filter and used to dilute proteins (50 μM, 2.2 ml), which were loaded into the sample cell and subjected to fast equilibration. Ligand stocks (5 μl, 100 mM) were diluted in filtered dialysis buffer (500 μl, 1 mM) and titrated against the protein at a stirring speed of 307 rpm. A total of 31 injections were performed, with an initial injection of 2 μl followed by 8 μl injections thereafter using 210 s time intervals. Control titrations were performed with ADP-ribose into buffer (31 injections) and buffer into buffer (10 injections). Data were analysed using NITPIC (initial parameters determined) [32,33], fitted to a one-site binding model using SEDPHAT [34], and visualised using GUSSI [32,35].

## Supplementary Material

Supplementary Figure S1-S10 and Table S1-S4

## Data Availability

All data supporting the findings are present in the main article and supplementary information. The DTX4 crystal structure is available at the Protein Data Bank with accession number 30XN [46]. Requests for additional data can be made to the corresponding authors.

## Open Access

Open access for this article was enabled by the participation of the University of Oxford in an all-inclusive Read & Publish agreement with Portland Press and the Biochemical Society under an agreement with JISC.

## Abbreviations

Δ*G*: change in Gibbs free energy
Δ*H*: change in enthalpy
Δ*S*: change in entropy
DTC: Deltex C-terminal
DTX: Deltex
His6: hexahistidine
IMAC: immobilised metal affinity chromatography
ITC: isothermal titration calorimetry
*K*_D_: dissociation constant
MAR: mono-ADP-ribose
NLRP4: NLR family pyrin domain containing 4
PAR: poly-ADP-ribose
RING: Really Interesting New Gene
RMSD: root mean square deviation
RNF4: RING finger protein 4
SEC: size-exclusion chromatography
SUMO: small ubiquitin-like modifier
TBK1: TANK binding kinase 1
TCEP: tris(2-carboxyethyl)phosphine
TEV: Tobacco Etch Virus
TRIM5α: tripartite motif 5α

## References

[1] Damgaard R.B. (2021) The ubiquitin system: from cell signalling to disease biology and new therapeutic opportunities. Cell Death Differ. 28, 423–426 33446876 10.1038/s41418-020-00703-w PMC7862391

[2] Ebadi P., Stratton C.M. and Olsen S.K. (2025) E3 ubiquitin ligases in signaling, disease, and therapeutics. Trends Biochem. Sci. 50, 960–976 40940201 10.1016/j.tibs.2025.07.009 PMC12435903

[3] Komander D. and Rape M. (2012) The ubiquitin code. Annu. Rev. Biochem. 81, 203–229 22524316 10.1146/annurev-biochem-060310-170328

[4] Swatek K.N. and Komander D. (2016) Ubiquitin modifications. Cell Res. 26, 399–422 27012465 10.1038/cr.2016.39 PMC4822133

[5] Wang L., Sun X., He J. and Liu Z. (2021) Functions and molecular mechanisms of Deltex family ubiquitin E3 ligases in development and disease. Front. Cell. Dev. Biol. 9, 706997 34513839 10.3389/fcell.2021.706997 PMC8424196

[6] Chastagner P., Rubinstein E. and Brou C. (2017) Ligand-activated Notch undergoes DTX4-mediated ubiquitylation and bilateral endocytosis before ADAM10 processing. Sci. Signal. 10, eaag2989 28611181 10.1126/scisignal.aag2989

[7] Cui J., Li Y.Y., Zhu L., Liu D., Zhou S.Y., Wang H.L.Y. et al. (2012) NLRP4 negatively regulates type I interferon signaling by targeting the kinase TBK1 for degradation via the ubiquitin ligase DTX4. Nat. Immunol. 13, 387–395 22388039 10.1038/ni.2239 PMC3760161

[8] Ahmed S.F., Buetow L., Gabrielsen M., Lilla S., Chatrin C., Sibbet G.J. et al. (2020) DELTEX2 C-terminal domain recognizes and recruits ADP-ribosylated proteins for ubiquitination. Sci. Adv. 6, eabc0629 32937373 10.1126/sciadv.abc0629 PMC7442474

[9] van Toorn M., Turkyilmaz Y., Han S., Zhou D., Kim H.S., Salas-Armenteros I. et al. (2022) Active DNA damage eviction by HLTF stimulates nucleotide excision repair. Mol. Cell. 82, 1343–1358 35271816 10.1016/j.molcel.2022.02.020 PMC9473497

[10] Rousseau A. and Bertolotti A. (2018) Regulation of proteasome assembly and activity in health and disease. Nat. Rev. Mol. Cell Biol. 19, 697–712 30065390 10.1038/s41580-018-0040-z

[11] Lin X., Sun B.C., Zhu D.W., Zhao X.L., Sun R., Zhang Y.H. et al. (2016) Notch4+cancer stem-like cells promote the metastatic and invasive ability of melanoma. Cancer Sci. 107, 1079–1091 27234159 10.1111/cas.12978 PMC4982579

[12] Liu M., Zhu K., Qian X. and Li W. (2016) Identification of miRNA/mRNA-negative regulation pairs in nasopharyngeal carcinoma. Med. Sci. Monit. 22, 2215–2234 27350400 10.12659/MSM.896047 PMC4928598

[13] Cui Y.T., Luo J., Bai N.F. and Yu Z.Y. (2023) Deltex E3 ubiquitin ligase 4 promotes thyroid cancer progression through stearoyl-CoA desaturase 1. Funct. Integr. Genomics 23, 23 37612343 10.1007/s10142-023-01215-9

[14] Xie Y., Li H., Luo X., Li H., Gao Q., Zhang L. et al. (2022) IBS 2.0: an upgraded illustrator for the visualization of biological sequences. Nucleic Acids Res. 50, W420–W426 35580044 10.1093/nar/gkac373 PMC9252815

[15] Madeira F., Madhusoodanan N., Lee J.H., Eusebi A., Niewielska A., Tivey A.R.N. et al. (2024) The EMBL-EBI Job Dispatcher sequence analysis tools framework in 2024. Nucleic Acids Res. 52, W521–W525 38597606 10.1093/nar/gkae241 PMC11223882

[16] Wong T.K.F., Ly-Trong N., Ren H., Demotte P. Banos H., Roger A.J. et al. (2026) IQ-TREE 3: phylogenomic inference software using complex evolutionary models. Mol. Biol. Evol. 43, 1–6 42085559 10.1093/molbev/msag117 PMC13191116

[17] Letunic I. and Bork P. (2024) Interactive Tree of Life (iTOL) v6: recent updates to the phylogenetic tree display and annotation tool. Nucleic Acids Res. 52, W78–W82 38613393 10.1093/nar/gkae268 PMC11223838

[18] Chatrin C., Gabrielsen M., Buetow L., Nakasone M.A., Ahmed S.F., Sumpton D. et al. (2020) Structural insights into ADP-ribosylation of ubiquitin by Deltex family E3 ubiquitin ligases. Sci. Adv. 6, eabc0418 32948590 10.1126/sciadv.abc0418 PMC7500938

[19] Zhu K., Suskiewicz M.J., Hlousek-Kasun A., Meudal H., Mikoc A., Aucagne V. et al. (2022) DELTEX E3 ligases ubiquitylate ADP-ribosyl modification on protein substrates. Sci. Adv. 8, eadd4253 36197986 10.1126/sciadv.add4253 PMC7615817

[20] Perrard J., Gao K., Ring K. and Smith S. (2025) Deltex and RING-UIM E3 ligases cooperate to create a ubiquitin-ADP-ribose hybrid mark on tankyrase, promoting its stabilization. Sci. Adv. 11, eadx7172 40901936 10.1126/sciadv.adx7172 PMC12407064

[21] Lacoursiere R.E., Upadhyaya K., Sidhu J.K., Siordia I.R. Bejan D.S., Cohen M.S. et al. (2025) RNF114 and RNF166 exemplify reader-writer E3 ligases that extend K11 polyubiquitin onto sites of MARUbylation. EMBO J. 44, 5993–6018 41039157 10.1038/s44318-025-00577-z PMC12583694

[22] Kloet M.S., Chatrin C., Mukhopadhyay R., van Tol B.D.M., Smith R., Rotman S.A. et al. (2025) Identification of RNF114 as ADPr-Ub reader through non-hydrolysable ubiquitinated ADP-ribose. Nat. Commun. 16, 6319 40634336 10.1038/s41467-025-61111-7 PMC12241653

[23] Djerir B., Marois I., Dubois J.C., Findlay S., Morin T., Senoussi I. et al. (2024) An E3 ubiquitin ligase localization screen uncovers DTX2 as a novel ADP-ribosylation-dependent regulator of DNA double-strand break repair. J. Biol. Chem. 300, 107545 38992439 10.1016/j.jbc.2024.107545 PMC11345397

[24] Kang B.G., Kang S.U., Kim J.J., Kwon J.S., Gagné J.P., Lee S.Y. et al. (2025) Proteome-wide microarray-based screening of PAR-binding proteins. Nucleic Acids Res. 53, gkaf300 40239998 10.1093/nar/gkaf300 PMC12000866

[25] Kelly M., Dietz C., Kasson S., Zhang Y., Holtzman M.J. and Kim I.K. (2024) Deltex family E3 ligases specifically ubiquitinate the terminal ADP-ribose of poly(ADP-ribosyl)ation. Biochem. Biophys. Res. Commun. 720, 150101 38749191 10.1016/j.bbrc.2024.150101 PMC11219154

[26] Münzker L., Kimani S.W., Fowkes M.M., Dong A.P., Zheng H., Li Y.J. et al. (2024) A ligand discovery toolbox for the WWE domain family of human E3 ligases. Commun. Biol. 7, 901 39048679 10.1038/s42003-024-06584-w PMC11269756

[27] Wang J.Y., Chitsaz F., Derbyshire M.K., Gonzales N.R., Gwadz M., Lu S.N. et al. (2023) The conserved domain database in 2023. Nucleic Acids Res. 51, D384–D388 36477806 10.1093/nar/gkac1096 PMC9825596

[28] Abramson J., Adler J., Dunger J., Evans R., Green T., Pritzel A. et al. (2024) Accurate structure prediction of biomolecular interactions with AlphaFold 3. Nature 630, 493–500 38718835 10.1038/s41586-024-07487-w PMC11168924

[29] Pike A.C., Rellos P., Niesen F.H., Turnbull A., Oliver A.W., Parker S.A. et al. (2008) Activation segment dimerization: a mechanism for kinase autophosphorylation of non-consensus sites. EMBO J. 27, 704–714 18239682 10.1038/emboj.2008.8 PMC2239268

[30] Buetow L., Gabrielsen M., Anthony N.G., Dou H., Patel A., Aitkenhead H. et al. (2015) Activation of a primed RING E3-E2-ubiquitin complex by non-covalent ubiquitin. Mol. Cell. 58, 297–310 25801170 10.1016/j.molcel.2015.02.017

[31] Wang Z.Z., Michaud G.A., Cheng Z.H., Zhang Y., Hinds T.R., Fan E.K. et al. (2012) Recognition of the iso-ADP-ribose moiety in poly(ADP-ribose) by WWE domains suggests a general mechanism for poly (ADP-ribosyl)ation-dependent ubiquitination. Genes Dev. 26, 235–240 22267412 10.1101/gad.182618.111 PMC3278890

[32] Keller S., Vargas C., Zhao H.Y., Piszczek G., Brautigam C.A. and Schuck P. (2012) High-precision isothermal titration calorimetry with automated peak-shape analysis. Anal. Chem. 84, 5066–5073 22530732 10.1021/ac3007522 PMC3389189

[33] Scheuermann T.H. and Brautigam C.A. (2015) High-precision, automated integration of multiple isothermal titration calorimetric thermograms: new features of NITPIC. Methods 76, 87–98 25524420 10.1016/j.ymeth.2014.11.024 PMC4380771

[34] Zhao H.Y., Piszczek G. and Schuck P. (2015) SEDPHAT—a platform for global ITC analysis and global multi-method analysis of molecular interactions. Methods 76, 137–148 25477226 10.1016/j.ymeth.2014.11.012 PMC4380758

[35] Brautigam C.A. (2015) Calculations and publication-quality illustrations for analytical ultracentrifugation data. Methods Enzymol. 562, 109–133 26412649 10.1016/bs.mie.2015.05.001

[36] Seyedarabi A., Sullivan J.A., Sasakawa C. and Pickersgill R.W. (2010) A disulfide driven domain swap switches off the activity of Shigella IpaH9.8 E3 ligase. FEBS Lett. 584, 4163–4168 20831869 10.1016/j.febslet.2010.09.006

[37] Rojas-Fernandez A., Plechanovova A., Hattersley N., Jaffray E., Tatham M.H. and Hay R.T. (2014) SUMO chain-induced dimerization activates RNF4. Mol. Cell. 53, 880–892 24656128 10.1016/j.molcel.2014.02.031 PMC3991395

[38] Herkules F., Yu C.H., Taylor A.B., Dougherty V., Weintraub S.T. and Ivanov D.N. (2022) Structural and functional asymmetry of RING trimerization controls priming and extension events in TRIM5alpha autoubiquitylation. Nat. Commun. 13, 7104 36402777 10.1038/s41467-022-34920-3 PMC9675739

[39] Savitsky P., Bray J., Cooper C.D.O., Marsden B.D., Mahajan P., Burgess-Brown N.A. et al. (2010) High-throughput production of human proteins for crystallization: the SGC experience. J. Struct. Biol. 172, 3–13 20541610 10.1016/j.jsb.2010.06.008 PMC2938586

[40] Verhoeven J.W. (1996) Glossary of terms used in photochemistry (IUPAC Recommendations 1996). Pure Appl. Chem. 68, 2223–2286 10.1351/pac199668122223

[41] Kabsch W. (2010) XDS. Acta Crystallogr. Sect. D Biol. Crystallogr. 66, 125–132 20124692 10.1107/S0907444909047337 PMC2815665

[42] Agirre J., Atanasova M., Bagdonas H., Ballard C.B., Baslé A., Beilsten-Edmands J. et al. (2023) The *CCP*4 suite: integrative software for macromolecular crystallography. Acta Crystallogr. D Struct. Biol. 79, 449–461 37259835 10.1107/S2059798323003595 PMC10233625

[43] McCoy A.J., Grosse-Kunstleve R.W., Adams P.D., Winn M.D., Storoni L.C. and Read R.J. (2007) *Phaser* crystallographic software. J. Appl. Crystallogr. 40, 658–674 19461840 10.1107/S0021889807021206 PMC2483472

[44] Murshudov G.N., Skubák P., Lebedev A.A., Pannu N.S., Steiner R.A., Nicholls R.A. et al. (2011) *REFMAC*5 for the refinement of macromolecular crystal structures. Acta Crystallogr. Sect. D Biol. Crystallogr. 67, 355–367 21460454 10.1107/S0907444911001314 PMC3069751

[45] Chen V.B., Arendall W.B., Headd J.J., Keedy D.A., Immormino R.M., Kapral G.J. et al. (2010) *MolProbity:* all-atom structure validation for macromolecular crystallography. Acta Crystallogr. Sect. D Biol. Crystallogr. 66, 12–21 20057044 10.1107/S0907444909042073 PMC2803126

[46] Fowkes M.M., Nair A. and Elkins J.M. (2026) Human DTX4 RING-DTC domains. Protein Data Bank Available from: https://www.rcsb.org/structure/unreleased/30XN

