# Supplementary material for: Structural and biophysical studies of human E3 ligase Deltex 4
Source: Biochem J. 2026 Oct 27;483(11):2241–54. doi: 10.1042/BCJ20260406 (PMC13714323; doi:10.1042/BCJ20260406)
Supplement: Supplementary file 1 [file BCJ-2026-0406_supp.pdf]

Supplementary information

Structural and biophysical studies of human E3 ligase Deltex 4

Milan M. Fowkes[1] and Jonathan M. Elkins[1]

[1]Centre for Medicines Discovery, Nuffield Department of Medicine, University of Oxford, NDM Research Building, Old Road Campus, Oxford, OX3 7FZ, U.K.

Correspondence: Jonathan M. Elkins (jon.elkins@cmd.ox.ac.uk) or Milan M. Fowkes (milan.fowkes@cmd.ox.ac.uk)

Table of Contents

Figures and Tables....................2

Supplementary Table 1. Summary of DTX4 RING-DTC constructs expressed in this study....................2

Supplementary Figure 1. Domain architecture and characterisation data for purified DTX4 RING-DTC domains....................6

Supplementary Table 2. Data collection and refinement statistics....................3

Supplementary Figure 2. Sequence alignment of RING-DTC domains of Deltex proteins and structural comparison between RING domains....................7

Supplementary Figure 3. Structure of the DTX4 domain-swapped dimer coloured by crystallographic temperature factor....................8

Supplementary Figure 4. Chromatographic analysis of the DTX4 RING-DTC domains...9

Supplementary Figure 5. Chromatographic analysis of the DTX4 RING-DTC domains with ADP-ribose....................10

Supplementary Table 3. Summary of DTX4 DTC constructs expressed in this study 4

Supplementary Figure 6. Domain architecture and characterisation data for purified DTX4 DTC domain....................11

Supplementary Figure 7. Control titrations for ITC experiments....................12

Supplementary Table 4. Summary of DTX1 and DTX2 DTC constructs expressed in this study....................4

Supplementary Figure 8. Domain architecture and characterisation data for purified DTX1 and DTX2 DTC domains....................13

Supplementary Figure 9. ITC analysis of DTX1 and DTX2 DTC domains with ADP......14

Supplementary Figure 10. ITC analysis of DTX4 RING-DTC domains with ADP-ribose and ADP....................15

References....................16

Figures and Tables

Supplementary Table 1. Summary of DTX4 RING-DTC constructs expressed in this study

DTX4 RING-DTC construct 1 (Gln383–Asp619)*

SMQAKKGKTPEEVLKKYLQKVRHPPDEDCTICMERLTAPSGYKGPQPTVKPDLVGKLSRCGHVYHI
YCLVAMYNNGNKDGSLQCPTCKTIYGVKTGTQPPGKMEYHLIPHSLPGHPDCKTIRIIYSIPPGIQ
GPEHPNPGKSFSARGFPRHCYLPDSEKGRKVLKLLLVAWDRRLIFAIGTSSTTGESDTVIWNEVHH
KTEFGSNLTGHGYPDANYLDNVLAELAAQGISEDSTAQEKD

DTX4 RING-DTC construct 2 (Gln383–Ser610)*

SMQAKKGKTPEEVLKKYLQKVRHPPDEDCTICMERLTAPSGYKGPQPTVKPDLVGKLSRCGHVYHI
YCLVAMYNNGNKDGSLQCPTCKTIYGVKTGTQPPGKMEYHLIPHSLPGHPDCKTIRIIYSIPPGIQ
GPEHPNPGKSFSARGFPRHCYLPDSEKGRKVLKLLLVAWDRRLIFAIGTSSTTGESDTVIWNEVHH
KTEFGSNLTGHGYPDANYLDNVLAELAAQGIS

DTX4 RING-DTC construct 3 (Gly387–Asp619)*

SMGKTPEEVLKKYLQKVRHPPDEDCTICMERLTAPSGYKGPQPTVKPDLVGKLSRCGHVYHIYCLV
AMYNNGNKDGSLQCPTCKTIYGVKTGTQPPGKMEYHLIPHSLPGHPDCKTIRIIYSIPPGIQGPEH
PNPGKSFSARGFPRHCYLPDSEKGRKVLKLLLVAWDRRLIFAIGTSSTTGESDTVIWNEVHHKTEF
GSNLTGHGYPDANYLDNVLAELAAQGISEDSTAQEKD

DTX4 RING-DTC construct 4 (Gly387–Ser610)*

SMGKTPEEVLKKYLQKVRHPPDEDCTICMERLTAPSGYKGPQPTVKPDLVGKLSRCGHVYHIYCLV
AMYNNGNKDGSLQCPTCKTIYGVKTGTQPPGKMEYHLIPHSLPGHPDCKTIRIIYSIPPGIQGPEH
PNPGKSFSARGFPRHCYLPDSEKGRKVLKLLLVAWDRRLIFAIGTSSTTGESDTVIWNEVHHKTEF
GSNLTGHGYPDANYLDNVLAELAAQGIS

*The residue numbers refer to the full length DTX4 sequence UniProt Q9Y2E6.

Supplementary Table 2. Data collection and refinement statistics

**PDB ID**	**30XN**
**Data Collection**	
Space group	$P6_522$
Cell constants:	
a, b, c (Å), α, β, γ (°)	109.1, 109.1, 222.0, 90, 90, 120
Resolution (Å)[a]	29.13-2.80 (2.95-2.80)
Unique observations[a]	20026 (2851)
Completeness (%)	99.9 (100.0)
Redundancy[a]	28.4 (29.7)
R_{merge}[a]	0.348 (6.17)
$\langle I/\sigma(I) \rangle$[a]	10.0 (0.8)
CC(1/2)	0.997 (0.480)
**Refinement**	
R_{work}, R_{free}	0.23, 0.29
Number of atoms	3561
r.m.s.d. (bonds) (Å)	0.006
r.m.s.d. (angles) (°)	1.65
Average B-factors (chain A protein, chain A ligand, chain B protein, chain B ligand)*	74, 71, 93, 104

[a] Values in parentheses indicate data for the highest resolution shell

r.m.s.d., root mean square deviation

* residue B-factors after TLS-parameterisation

Supplementary Table 3. Summary of DTX4 DTC constructs expressed in this study

DTX4 DTC construct 1 (Lys468–Asp619)*

SMKTIYGVKTGTQPPGKMEYHLIPHSLPGHPDCKTIRIIYSIPPGIQGPEHPNPGKSFSARGFPRH
CYLPDSEKGRKVLKLLLVAWDRRLIFAIGTSSTTGESDTVIWNEVHHKTEFGSNLTGHGYPDANYL
DNVLAELAAQGISEDSTAQEKD

DTX4 DTC construct 2 (Lys468–Ser610)*

SMKTIYGVKTGTQPPGKMEYHLIPHSLPGHPDCKTIRIIYSIPPGIQGPEHPNPGKSFSARGFPRH
CYLPDSEKGRKVLKLLLVAWDRRLIFAIGTSSTTGESDTVIWNEVHHKTEFGSNLTGHGYPDANYL
DNVLAELAAQGIS

DTX4 DTC construct 3 (Lys474–Asp619)*

SMKTGTQPPGKMEYHLIPHSLPGHPDCKTIRIIYSIPPGIQGPEHPNPGKSFSARGFPRHCYLPDS
EKGRKVLKLLLVAWDRRLIFAIGTSSTTGESDTVIWNEVHHKTEFGSNLTGHGYPDANYLDNVLAE
LAAQGISEDSTAQEKD

DTX4 DTC construct 4 (Lys474–Ser610)*

SMKTGTQPPGKMEYHLIPHSLPGHPDCKTIRIIYSIPPGIQGPEHPNPGKSFSARGFPRHCYLPDS
EKGRKVLKLLLVAWDRRLIFAIGTSSTTGESDTVIWNEVHHKTEFGSNLTGHGYPDANYLDNVLAE
LAAQGIS

*The residue numbers refer to the full length DTX4 sequence UniProt Q9Y2E6.

Supplementary Table 4. Summary of DTX1 and DTX2 DTC constructs expressed in this study

DTX1 DTC construct (Lys472–Ala620)*

SMKAIYGEKTGTQPPGKMEFHLIPHSLPGFPDTQTIRIVYDIPTGIQGPEHPNPGKKFTARGFPRH
CYLPNNEKGRKVLRLLITAWERRLIFTIGTSNTTGESDTVVWNEIHHKTEFGSNLTGHGYPDASYL
DNVLAELTAQGVSEAAAKA

DTX2 DTC construct (Lys473–Gln622)**

SMKTIYGEKTGTQPQGKMEVLRFQMSLPGHEDCGTILIVYSIPHGIQGPEHPNPGKPFTARGFPRQ
CYLPDNAQGRKVLELLKVAWKRRLIFTVGTSSTTGETDTVVWNEIHHKTEMDRNITGHGYPDPNYL
QNVLAELAAQGVTEDCLEQQ

*The residue numbers refer to the full length DTX1 sequence UniProt Q86Y01

**The residue numbers refer to the full length DTX2 sequence UniProt Q86UW9

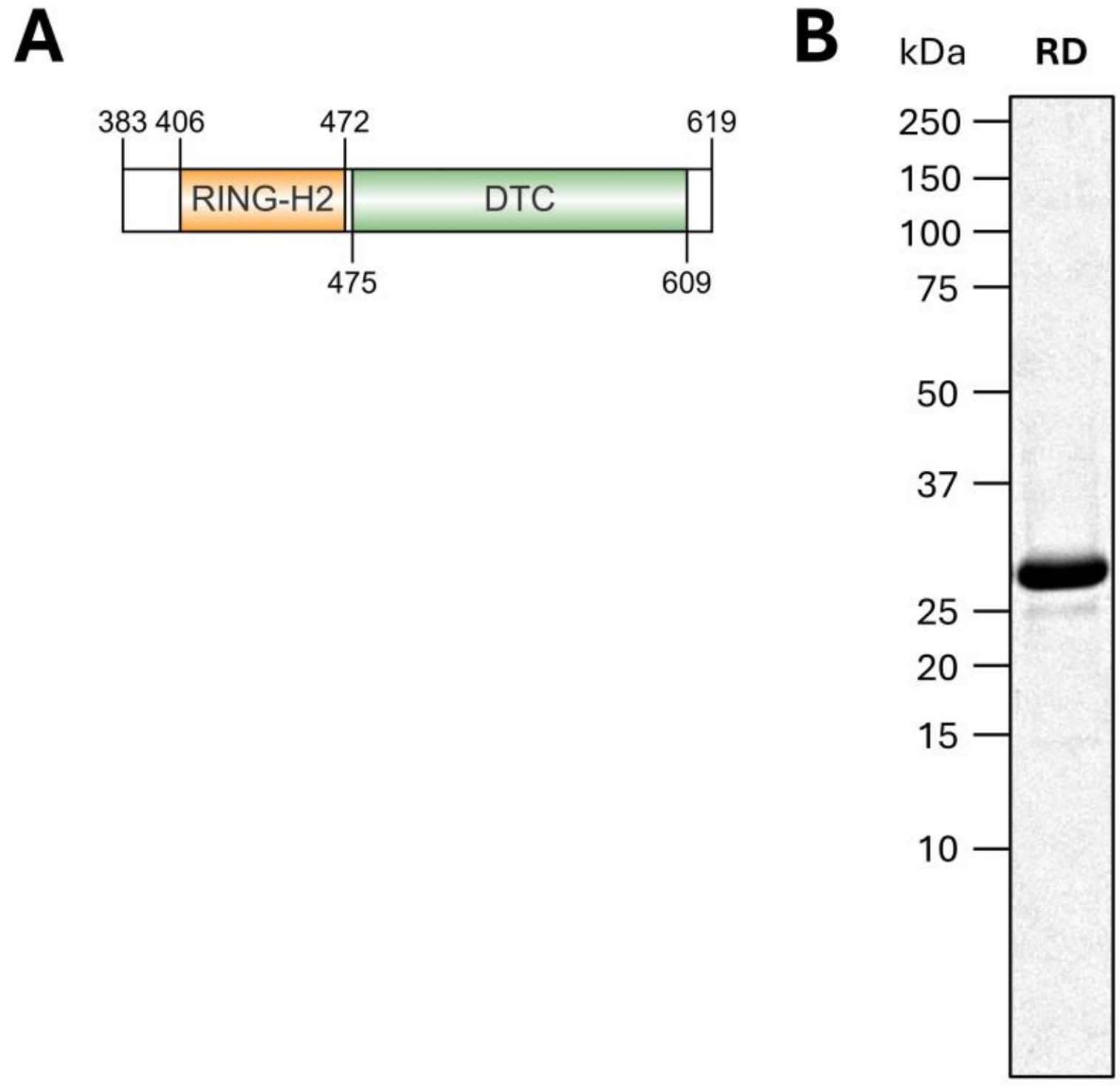

Supplementary Figure 1. Domain architecture and characterisation data for purified DTX4 RING-DTC domains

(**A**) Domain architecture of human DTX4 RING-DTC construct 1 used in this study, with domain boundaries derived by analysis against the NCBI conserved domain database [1]. (**B**) Representative Coomassie stained SDS-PAGE of purified DTX4 RING-DTC domains (RD) protein.

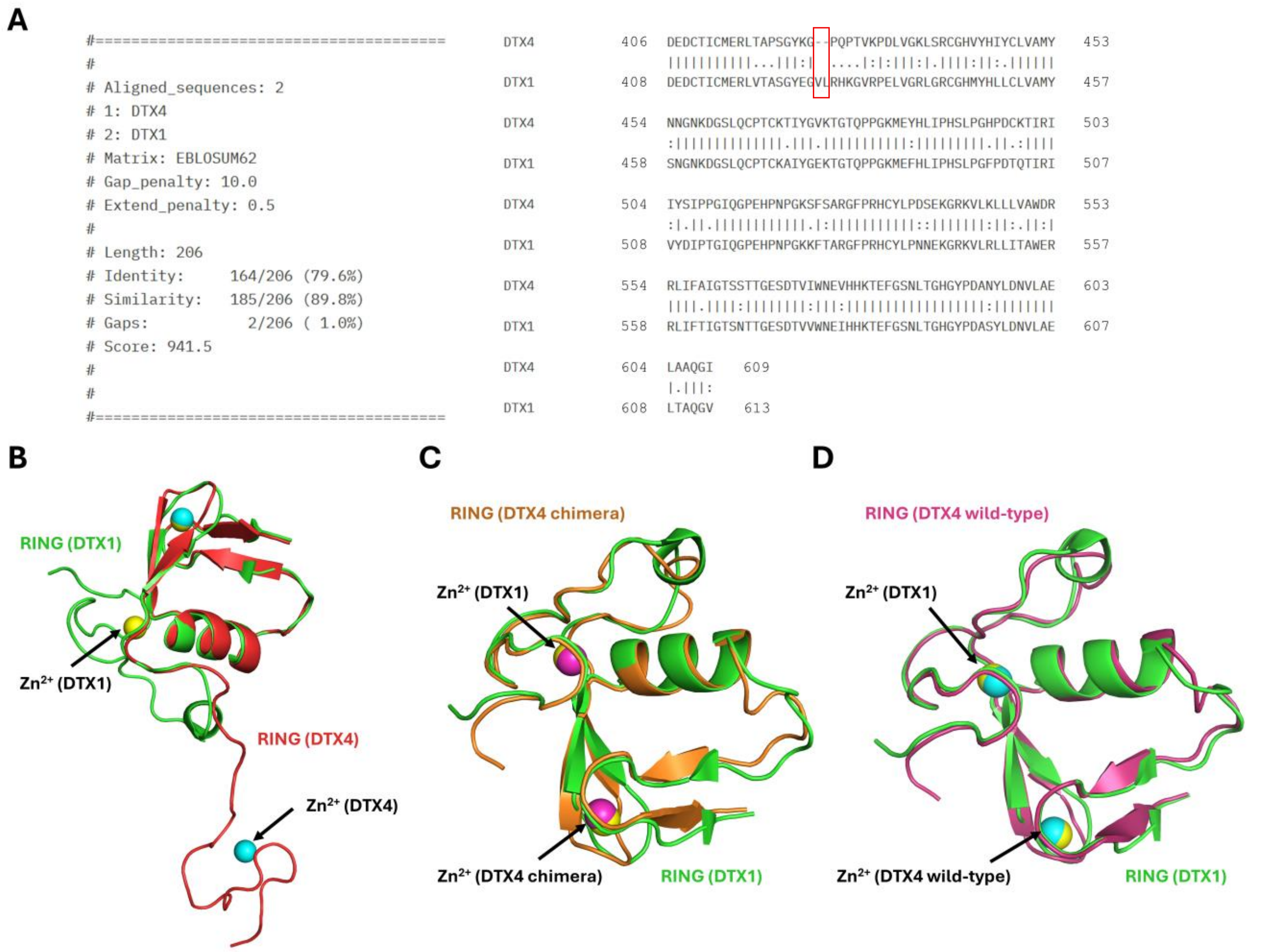

Supplementary Figure 2. Sequence alignment of RING-DTC domains of Deltex proteins and structural comparison between RING domains

(**A**) Pairwise sequence alignment of the RING-DTC domains of DTX4 and DTX1 using EMBOSS Water *via* the EMBL-EBI Job Dispatcher tool [2]. Residues absent in DTX4 are highlighted in red. (**B**) Superposition of the RING domains of DTX4 and DTX1 (PDB 6Y5N). (**C, D**) Superposition of the RING domain of DTX1 (PDB 6Y5N) with (**C**) the structure of the DTX1-DTX4 chimera consisting of the DTX4 RING domain with Val-Leu residues from DTX1 inserted into the 422–428 loop, and (**D**) the wild-type DTX4 RING domain structure without the additional two residues. Both chimera and wild-type structural predictions were generated by the Alphafold 3 server.

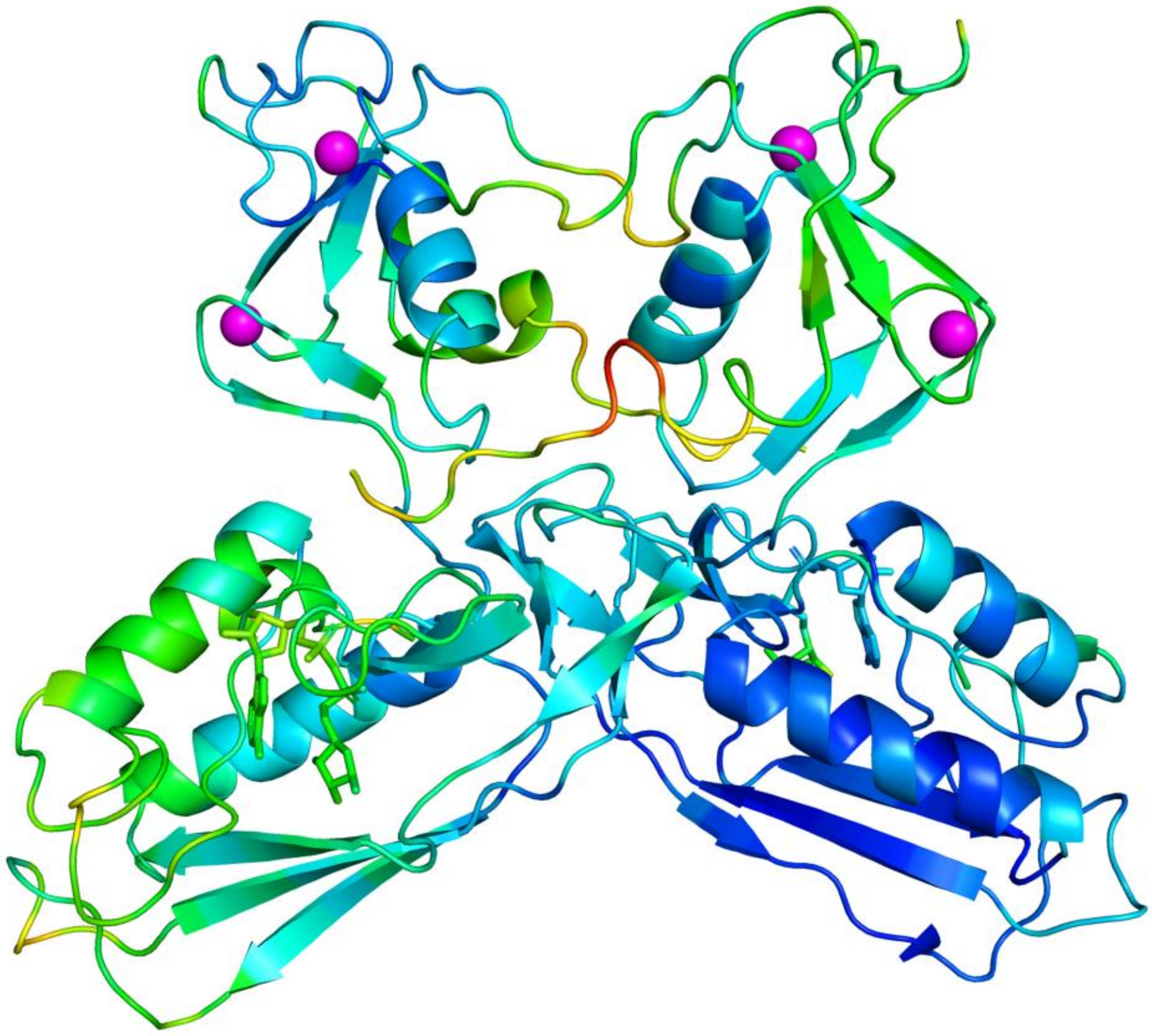

Supplementary Figure 3. Structure of the DTX4 domain-swapped dimer coloured by crystallographic temperature factor

The structure was initially refined without the use of TLS parameters and is shown in a similar orientation to Figure 2B. The B-factors are coloured from low (blue) to high (red) using a rainbow colour gradient. Zinc ions are depicted as magenta spheres.

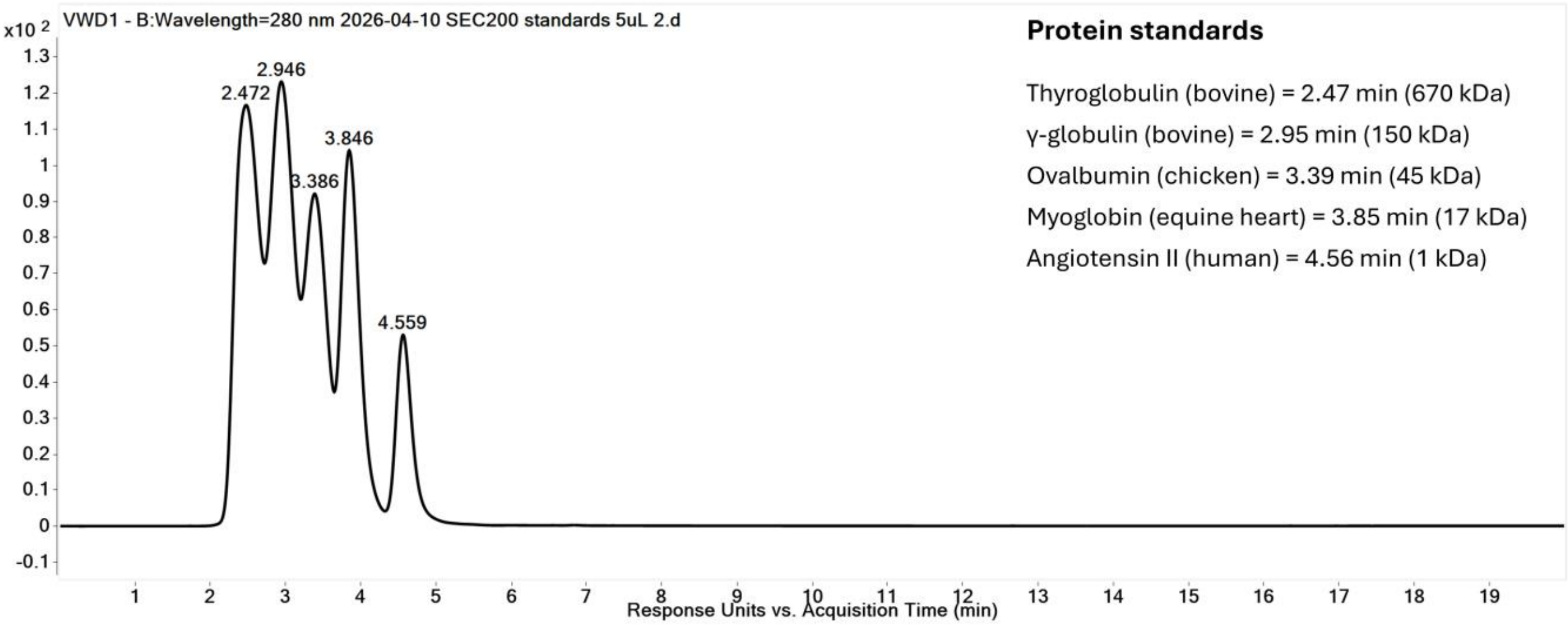

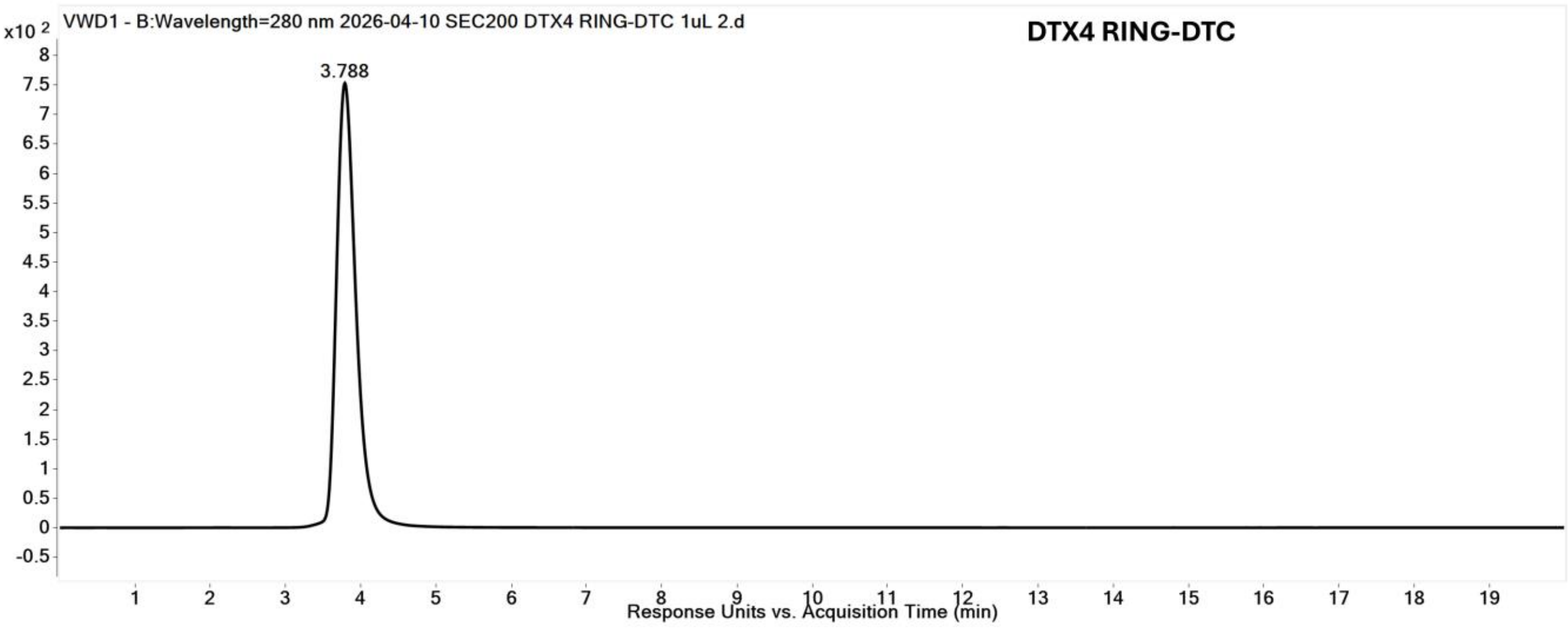

Supplementary Figure 4. Chromatographic analysis of the DTX4 RING-DTC domains

Analytical size exclusion chromatogram of the DTX4 RING-DTC domain construct 1 (bottom) compared to a set of protein standards (top).

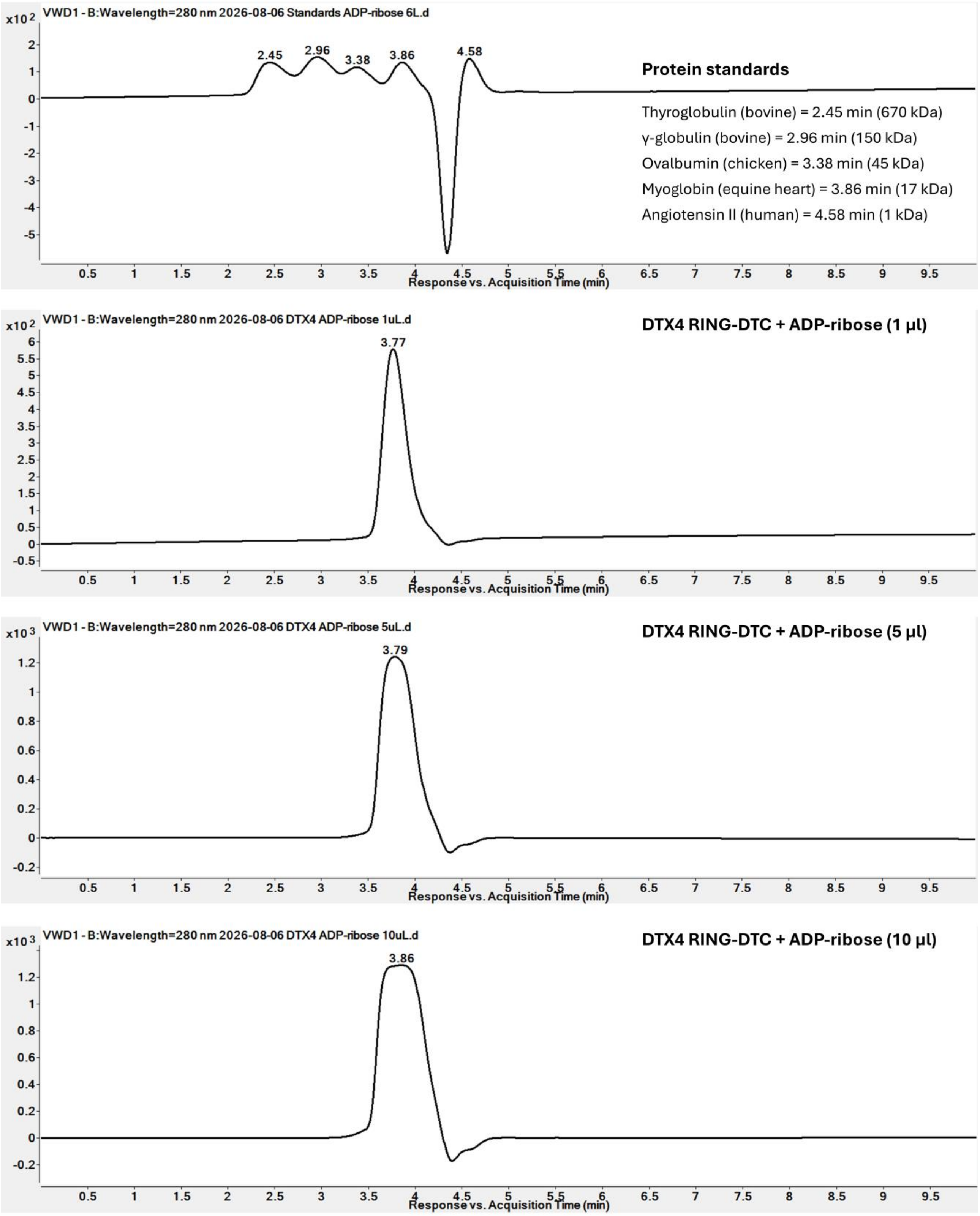

Supplementary Figure 5. Chromatographic analysis of the DTX4 RING-DTC domains with ADP-ribose

Analytical size exclusion chromatogram of the DTX4 RING-DTC domain construct 1 with ADP-ribose (bottom panels) compared to a set of protein standards (top panel).

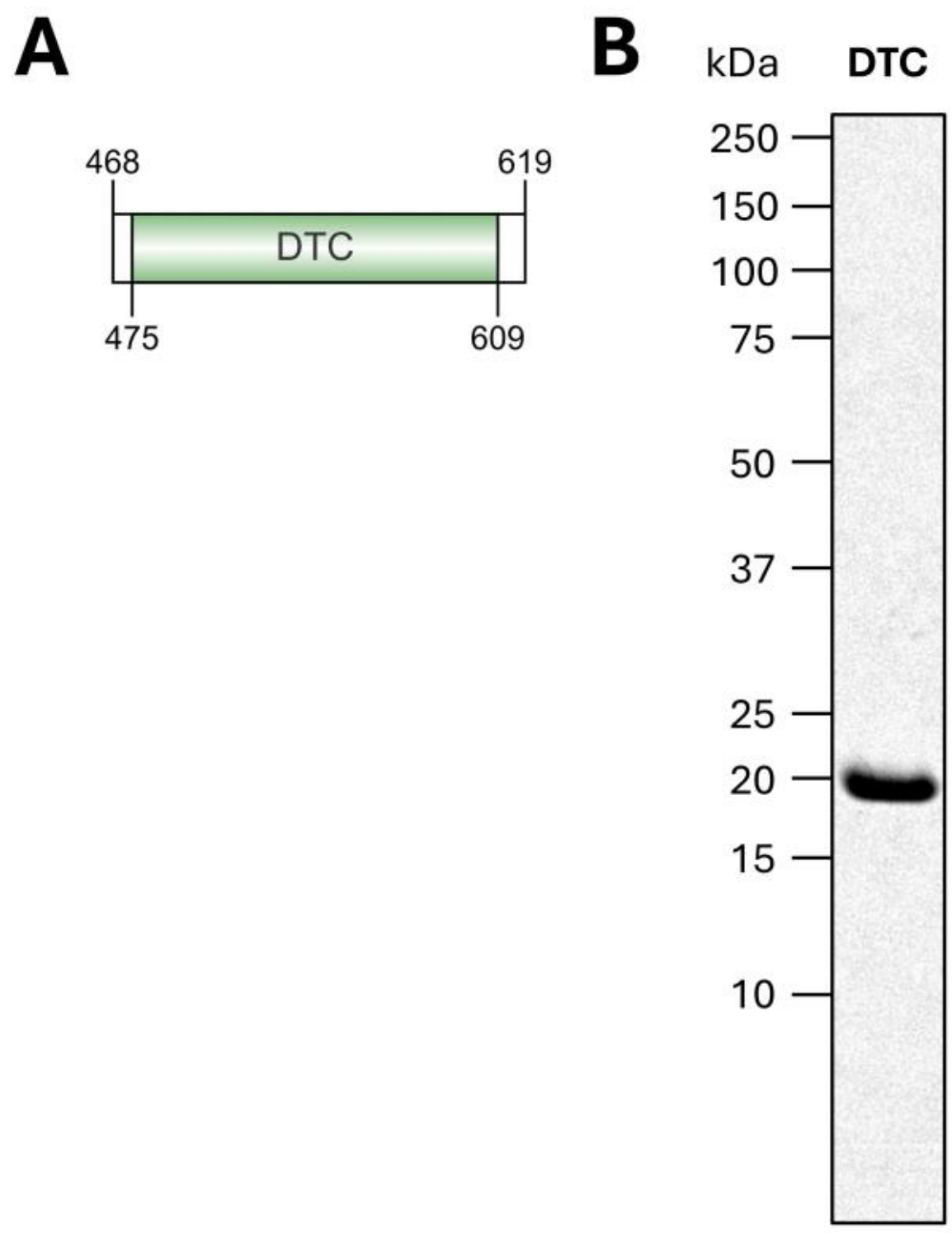

Supplementary Figure 6. Domain architecture and characterisation data for purified DTX4 DTC domain

(**A**) Domain architecture of human DTX4 DTC domain construct 1 used in this study, with domain boundaries derived by analysis against the NCBI conserved domain database [1]. (**B**) Representative Coomassie stained SDS-PAGE of purified DTX4 DTC domain protein.

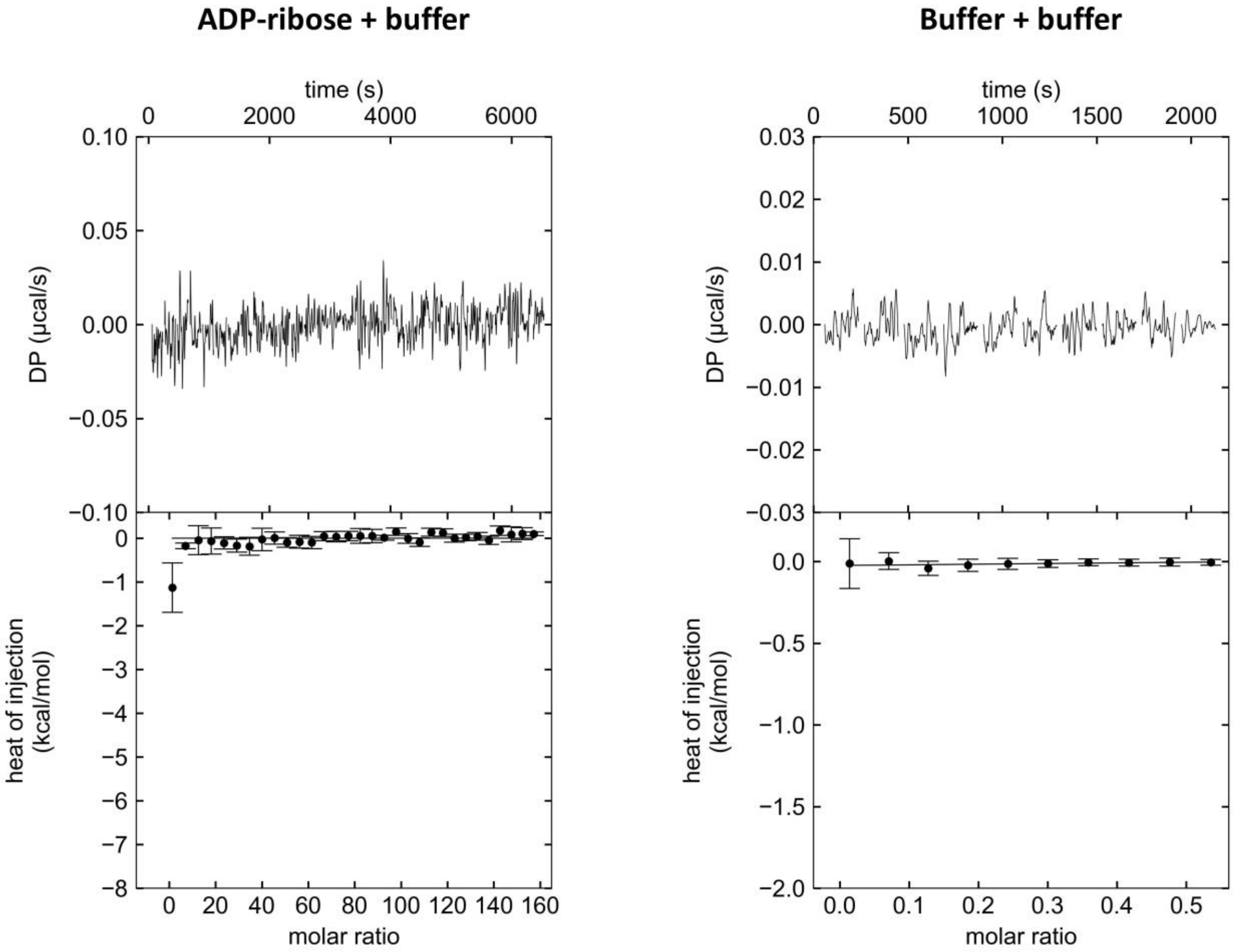

Supplementary Figure 7. Control titrations for ITC experiments

Isothermal titration calorimetry data for injections of ADP-ribose into buffer and buffer into buffer at 298 K. Data was processed with NITPIC [3, 4] and visualised with Gussi [5].

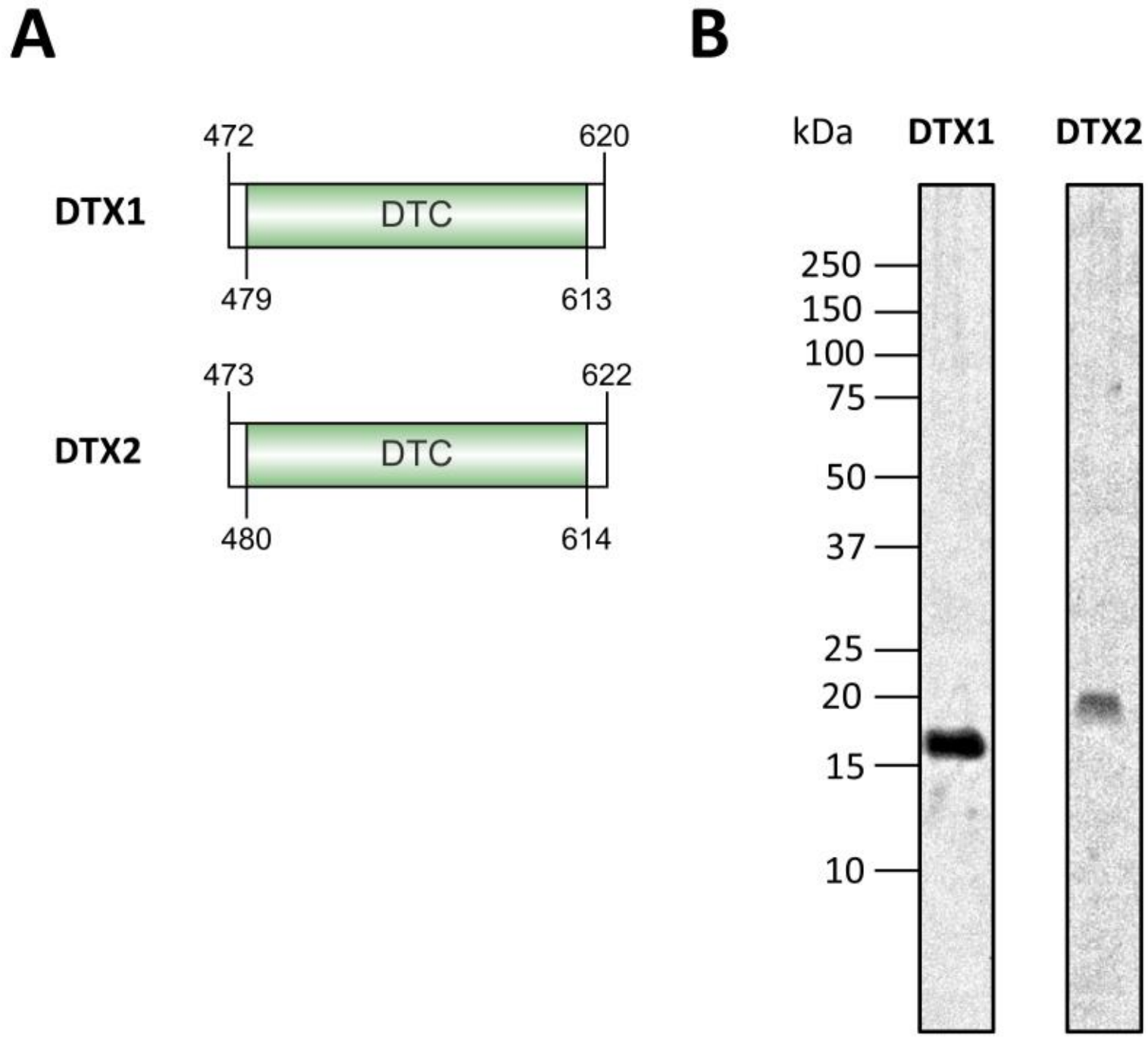

Supplementary Figure 8. Domain architecture and characterisation data for purified DTX1 and DTX2 DTC domains

(**A**) Domain architecture of human DTX1 and DTX2 DTC domain constructs used in this study, with domain boundaries determined by analysis against the NCBI conserved domain database [1]. (**B**) Representative Coomassie stained SDS-PAGE of purified DTX1 and DTX2 DTC domain proteins.

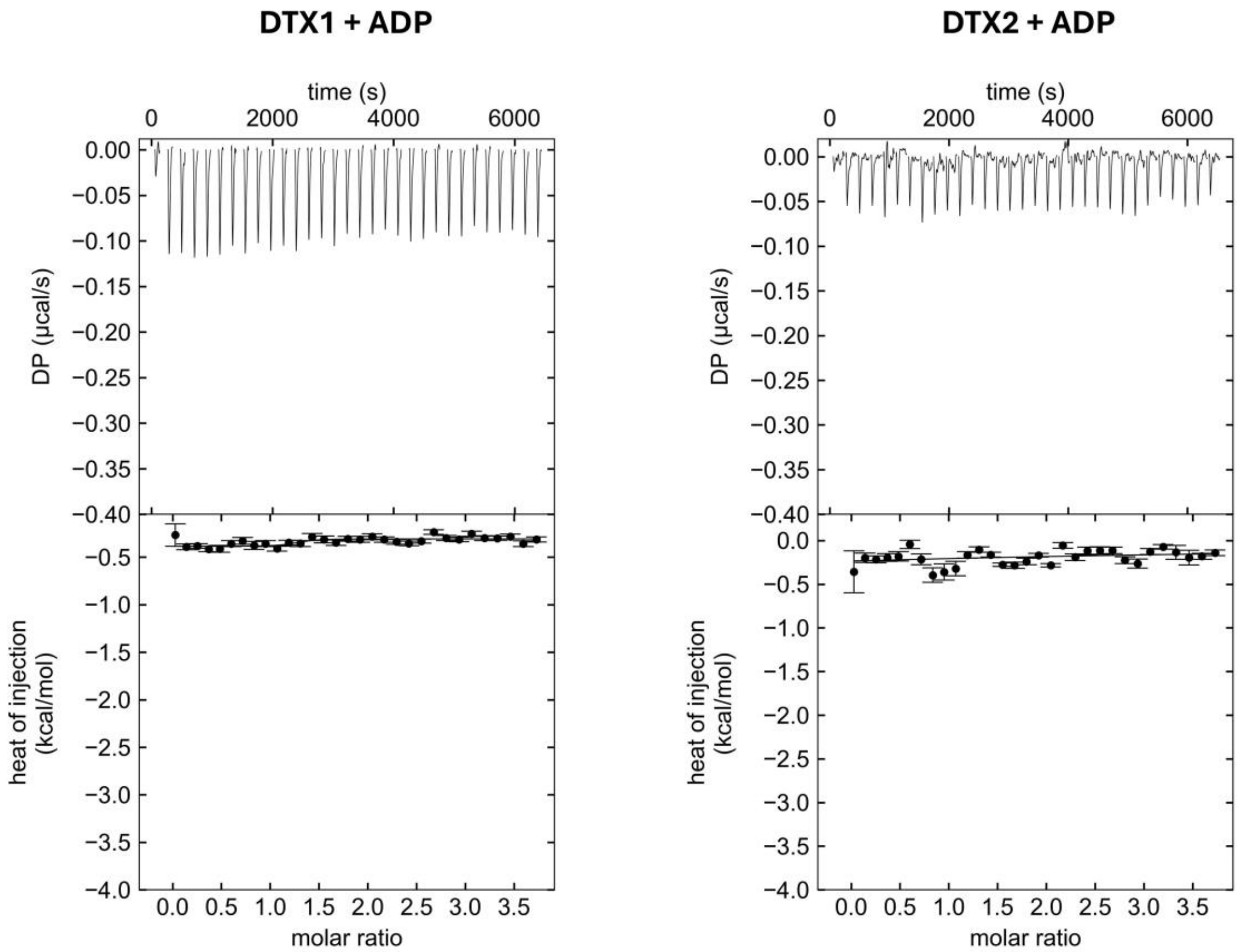

Supplementary Figure 9. ITC analysis of DTX1 and DTX2 DTC domains with ADP

Isothermal titration calorimetry data for DTX1 and DTX2 DTC domains with ADP. ADP was injected multiple times into a solution of DTX1 or DTX2 protein at 298 K. Data was processed with NITPIC [3, 4] and visualised with Gussi [5].

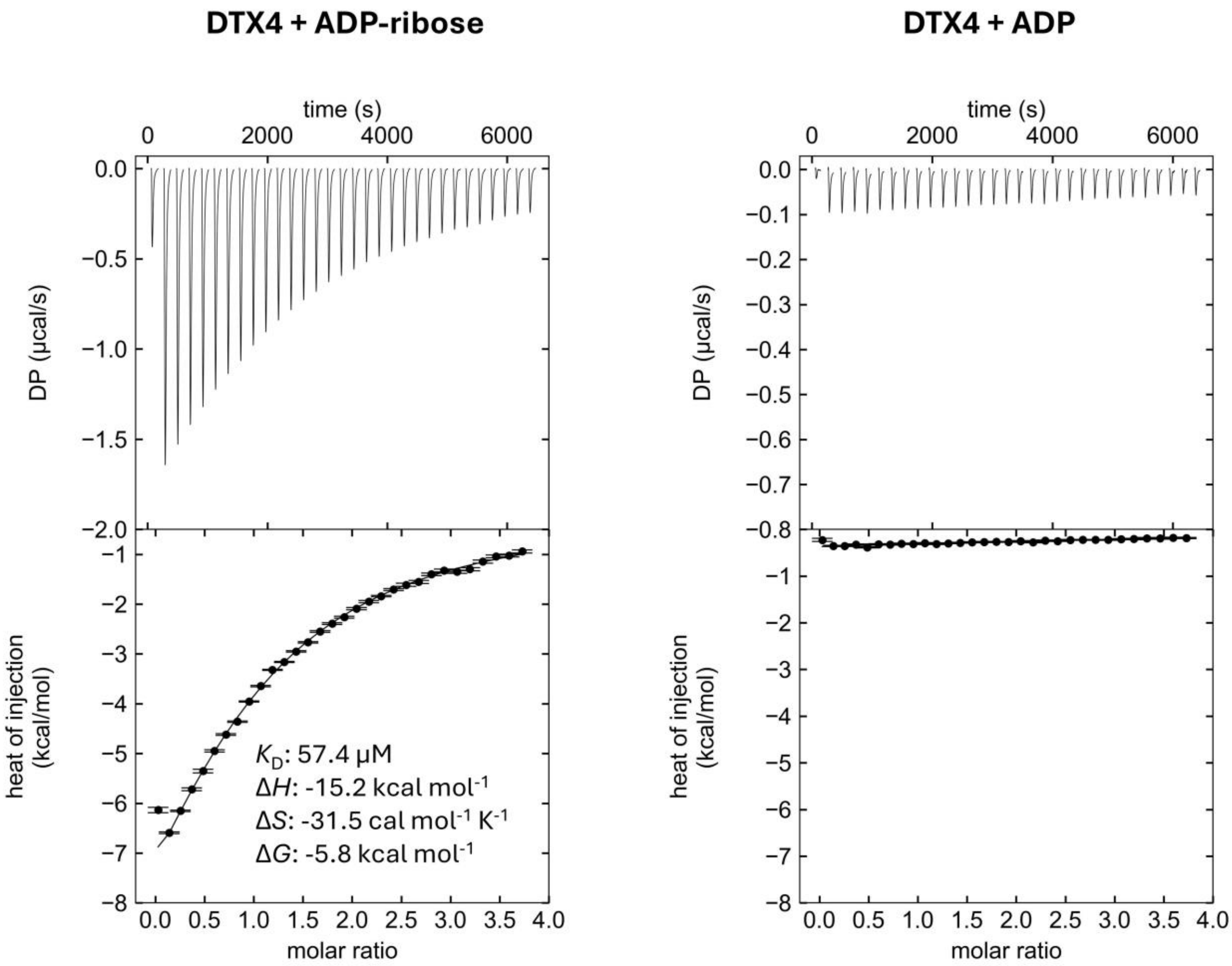

Supplementary Figure 10. ITC analysis of DTX4 RING-DTC domains with ADP-ribose and ADP

Isothermal titration calorimetry data for DTX4 RING-DTC domains with ADP-ribose or ADP. The ADP-ribose or ADP was injected multiple times into a solution of the DTX4 RING-DTC domains protein. Data was processed with NITPIC [3, 4] fitted to a one-site binding model using SEDPHAT [6] and visualised with Gussi [5].

References

1 Wang, J. Y., Chitsaz, F., Derbyshire, M. K., Gonzales, N. R., Gwadz, M., Lu, S. N., et al. (2023) The conserved domain database in 2023. Nucleic Acids Res. **51**, D384–D388 36477806 10.1093/nar/gkac1096

2 Madeira, F., Madhusoodanan, N., Lee, J. H., Eusebi, A., Niewielska, A., Tivey, A. R. N., et al. (2024) The EMBL-EBI Job Dispatcher sequence analysis tools framework in 2024. Nucleic Acids Res. **52**, W521–W525 38597606 10.1093/nar/gkae241

3 Keller, S., Vargas, C., Zhao, H. Y., Piszczek, G., Brautigam, C. A. and Schuck, P. (2012) High-Precision Isothermal Titration Calorimetry with Automated Peak-Shape Analysis. Anal. Chem. **84**, 5066–5073 22530732 10.1021/ac3007522

4 Scheuermann, T. H. and Brautigam, C. A. (2015) High-precision, automated integration of multiple isothermal titration calorimetric thermograms: New features of NITPIC. Methods. **76**, 87–98 25524420 10.1016/j.ymeth.2014.11.024

5 Brautigam, C. A. (2015) Calculations and Publication-Quality Illustrations for Analytical Ultracentrifugation Data. Methods Enzymol. **562**, 109–133 26412649 10.1016/bs.mie.2015.05.001

6 Zhao, H. Y., Piszczek, G. and Schuck, P. (2015) SEDPHAT - A platform for global ITC analysis and global multi-method analysis of molecular interactions. Methods. **76**, 137–148 25477226 10.1016/j.ymeth.2014.11.012
